# A Digital Patient Portal for Patients With Multiple Sclerosis

**DOI:** 10.3389/fneur.2020.00400

**Published:** 2020-05-22

**Authors:** Isabel Voigt, Martin Benedict, Marcel Susky, Tim Scheplitz, Sophie Frankowitz, Raimar Kern, Olaf Müller, Hannes Schlieter, Tjalf Ziemssen

**Affiliations:** ^1^Department of Neurology, Center of Clinical Neuroscience, University Hospital Carl Gustav Carus, Technical University of Dresden, Dresden, Germany; ^2^Chair of Wirtschaftsinformatik, Especially Systems Development, Faculty of Business and Economics, Technical University of Dresden, Dresden, Germany; ^3^MedicalSyn GmbH, Dresden, Germany; ^4^Carus Consilium GmbH, Dresden, Germany

**Keywords:** digital technology, eHealth, patient engagement, patient portals, clinical pathway, neurological disease, chronic disease, multiple sclerosis

## Abstract

**Background:** Multiple Sclerosis is a chronic inflammatory disease of the central nervous system that requires a complex, differential, and lifelong treatment strategy, which involves high monitoring efforts and the accumulation of numerous medical data. A fast and broad availability of care, as well as patient-relevant data and a stronger integration of patients and participating care providers into the complex treatment process is desirable. The aim of the ERDF-funded project “Integrated Care Portal Multiple Sclerosis” (IBMS) was to develop a pathway-based care model and a corresponding patient portal for MS patients and health care professionals (HCPs) as a digital tool to deliver the care model.

**Methods:** The patient portal was created according to a patient-centered design approach which involves both the patients' and the professionals' view. Buurmann's five iterative phases were integrated into a design science research process. A problem analysis focusing on functions and user interfaces was conducted through surveys and workshops with MS patients and HCPs. Based on this, the patient portal was refined and a prototype of the portal was implemented using an agile software development strategy.

**Results:** HCPs and patients already use digital hardware and are open to new technologies. Nevertheless, they desire improved (digital) communication and coordination between care providers. Both groups require a number of functions for the patient portal, which were implemented in the prototype. Usability tests with patients and HCPs are planned to consider whether the portal is deemed as usable, acceptable as well as functional to prepare for any needed ameliorations.

**Discussion:** After testing the patient portal for usability, acceptability, and functionality, it will most likely be a useful and high-quality electronic health (eHealth) tool for patient management from day care to telerehabilitation. It implements clinical pathways in a manner which is comprehensible for patients. Future developments of the patient portal modules could include additional diseases, the integration of quality management and privacy management tools, and the use of artificial intelligence to personalize treatment strategies.

## Introduction

Multiple Sclerosis (MS) is a chronic inflammatory, neurodegenerative disease of the central nervous system which leads to a wide range of neurological deficits. It is typically diagnosed in young adult patients between the ages of 20 and 40, and, for the most part, it initially follows a relapsing course. The highly individual symptoms often include fatigue, visual and bladder disorders, pain, spasticity, mobility, and sexual restrictions, as well as psychological disorders such as depression (1, 2), which is why it is popularly referred to as the “disease of a thousand faces” (3). MS patients therefore need to be treated by multi-professional, inter-institutional, and cross-sectoral health care teams, e.g., MS specialists, neurologists, and general practitioners as well as specific specialists and therapists (4, 5). The often decades-long, unpredictable disease course requires ongoing and long-term monitoring, assessment, and management, preferably with digital applications for health care professionals (HCPs) as well as patients (6, 7).

Digital applications are part of the digital transformation in healthcare, which will see the integration of technologies such as advanced analytics, machine learning, and artificial intelligence (8). Digital transformation in healthcare can lead to improvements in diagnosis, prevention, and therapy. It enables HCPs to apply an evidence-based approach to improve clinical decision-making (8, 9). Further examples are the provision of comprehensive information and the rapid exchange of reports and information between patients, experts, and medical centers. Especially in the case of complex, unpredictable, and chronically progressive diseases such as MS, digitalization and electronic health (eHealth) systems can help to better diagnose, monitor, and thus optimally treat individual patients (6).

In the context of improving the treatment of patients, concepts of patient-centered care and shared decision-making must also be mentioned as features of a high-quality health care (10). The Institute of Medicine defines patient-centered care as: “Providing care that is respectful of, and responsive to, individual patient preferences, needs and values, and ensuring that patient values guide all clinical decisions” (11). Patients involved in the treatment process show higher treatment adherence and better treatment outcomes (12–14). In contrast to a role of patients limited to a period of time, chronically ill patients (including MS patients) must play a greater role in shaping their treatment and become experts of their individual care (14, 15). The mostly younger MS patients have a high digital affinity and a high competence in the indexing and use of eHealth services to promote their own patient competence (16–20). To make involvement possible, patients should have access to, as well as understanding of, their treatment plans and context-sensitive information concerning their health status. This means explaining to the patient in a way that is easy for the layperson to understand which treatment steps are being carried out including why, when, and how with regards to their particular phase of illness. Through this, patients get involved in their treatment process and thus, become co-deciders of their treatment. The access to such information can be supported by patient-centered health information technologies, such as patient portals (10). Patient portals are increasingly showing their potential as cost-effective methods that can both improve patients' quality of life and serve as useful tools for patient participation (21). They are also ascribed potential for improving the quality of care (22–24). In general, patient portals have been little used in the German health care system for care management and particularly for involvement of patients so far (25). Especially in the area of clinics, they have mostly been used as information kiosks. This provides a more informational approach for patients without requiring their participation.

In our research, a patient portal for MS patients and HCPs is being developed in the course of inter-organizational MS care. It allows the patient to follow the course of treatment and to correspond with service providers based on the course of treatment (26). The authors aim to introduce patient-centered MS care by implementing a pathway-based care model and by using digital technologies. The electronic patient portal provides personalized information technology (IT) assisted clinical pathways (c.f. section Theory) on the basis of a Fast Healthcare Interoperability Resource (FHIR)-based architecture (FHIR is an interoperability standard for sharing data between application systems in healthcare) (26).

This paper describes the conception of the patient portal based on current knowledge of patient portals and clinical pathways, as well as an existing documentation system (Multiple Sclerosis Documentation System, MSDS^3D^) (27, 28) and a specifically developed MS case record (c.f. section Theory). In order to successfully implement the patient portal, end users' needs and concerns were taken into account (20, 29). Based on a user-centered design approach (30–32) and a patient-centered participatory design process (21, 33), surveys and workshops with MS patients and HCPs were conducted. The results were incorporated into the development of the portal. The paper provides insight into the functional demands of patients as well as HCPs and shows how these demands can be operationalized in a digitalized MS care model. It describes a technological model for a patient portal, which implements this digitalized MS care model.

The portal named “Integrated Multiple Sclerosis Care Portal”—IBMS—was collaboratively developed by HCPs of the Multiple Sclerosis Center at the Carl Gustav Carus University Hospital and developers of the Chair of Wirtschaftsinformatik, especially System Development at the Technical University of Dresden as well as with the help of MedicalSyn GmbH and Carus Consilium GmbH.

## Theory

### Patient Portals and Clinical Pathways

Digital patient portals serve as the basis for patient involvement and for the IT support of MS treatment processes. The recommendations of Van den Bulck et al. concerning patient portal design should be mentioned here as examples of the current state of research: they recommend providing a clinical summary to the patient after each visit, secure messaging between patient and provider, the ability to view, download, and transmit personal health record data, patient specific education, patient reminders for preventative services, and medication reconciliation (10).

**Figure 1:**
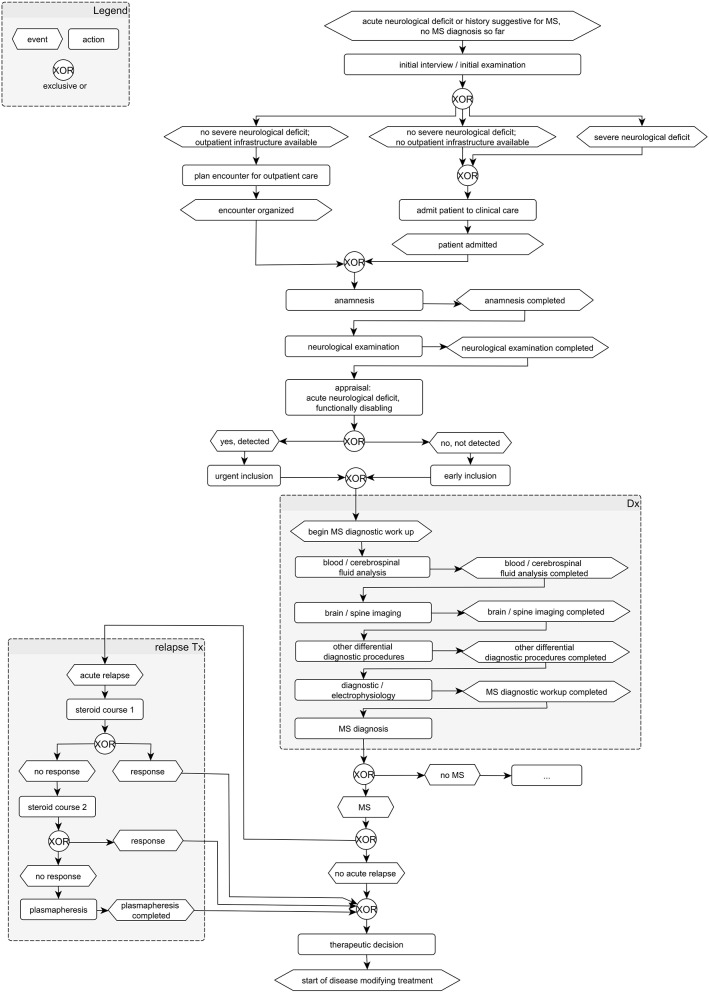
Example for MS pathway model.

Similar to a checklist in the pilot cockpit, these aspects and the diagnostic and therapeutic procedure can be optimized using defined clinical pathways. Clinical pathways are particularly suitable for the seamless care of chronically ill patients across various health sectors. They describe the entire path of patients during care and unite the multidisciplinary setting, the local conditions, and the current state of evidence research (see Figure 1). The focus is on the advance planning of concrete steps of action that are linked to temporal or defined changes in condition (26). In this way, clinical pathways define goals and milestones of care and support the joint decision-making of patients and the multidisciplinary care team involved. Furthermore, patients get more clarity as to which phase of the disease they are in and what current disease activity they have. They are put in a position to contribute to the improvement or maintenance of their state of health by comprehensible situation-oriented recommendations for action. This is intended to strengthen patient competence and intensify the HCP-patient relationship without additional effort on the part of the HCP (26).

Moreover, HCPs were supported in the organization and quality management of care. A consensus-based standardized management path to integrated MS care can contribute as a basis for the development of innovative inter-organizational processes (34). A consensus MS path serves not only as a structure for process organization and quality assurance of MS treatment, but also as an instrument for collecting structured multidimensional data on individual cases of MS in order to develop personalized strategies for MS treatment management (35). These pathways serve various purposes within MS care:

1. **MS care coordination:** The representation of a care model can be done by providing graphical models with dynamic aspects (e.g., the flow of the patient) as well as static aspects (e.g., document structures) (36).
2. **Documentation of patient status:** clinical pathways serve as a tool for managing the patient encounters and the documentation of the current patient status.
3. **Development of patient pathways:** clinical pathways are used and transformed into patient pathways based on patient-specific documentation.
4. **Identification of information flows:** clinical pathways are used to identify information flows for implementing necessary technological measures.

### Multiple Sclerosis Documentation System MSDS^3D^

To manage MS care in a high-quality manner, a certain amount of clinical data is necessary. The relevant data needed, e.g., clinical data, laboratory values, results of magnetic resonance imaging (MRI), and questionnaires (37), are gathered, in the project described here, using a disease-specific software: the multidimensional Multiple Sclerosis Documentation System (MSDS^3D^), which was developed by the eHealth project group at the University Hospital Dresden and has been continued by MedicalSyn GmbH since 2014. MSDS^3D^ supports patients, nurses, and HCPs (38) in carrying out complex processes such as therapy management (39–41) and it forms the control center for the medical care providers across all institutions and sector boundaries. For clinical data acquisition, the personal and individual circumstances of the participating patients are recorded using tablet-based online questionnaires. Interaction with the patients takes place either via online multi-touch systems, e.g., a touch screen or touch pad as an interactive patient terminal, or via mobile devices, e.g., the patient's smartphone (42, 43). In the MSDS^3D^ system, the recorded data is integrated promptly and actively into the individual treatment process of each patient and can be networked according to standardized clinical treatment paths. MSDS^3D^ regularly reminds the treating HCP of important laboratory or image controls that have to be performed for certain immunotherapies in order to generate large drug-specific real world datasets for specific disease-modifying drugs (44–48).

### MS Case Record

The portal also integrates a cross-institutional MS case record which can be accessed by various HCPs in MS care and by patients. Case records typically integrate clinical documentation systems and HCP document systems using electronic interfaces. They are commonly used to implement cross-institutional information exchange (49). The advantage of a case record is that, above all, new actors involved in care and treatment gain immediate insight into all relevant patient and treatment data (50). The prerequisite for storing and viewing the data is the patient's consent, which must always be obtained in writing in accordance with the currently applicable data protection regulations (in Europe: GDPR) (47). The electronic MS case record contains all MS-related information on the patient, and it is mostly used by providers for diagnosis and treatment. A central part of a MS case record is the metadata assigned to the containing documents. A comprehensive specification of this data is crucial both for information provision as well as for information retrieval. It harmonizes different terminologies from different participating systems (semantic interoperability). Consequently, it must represent the terminology of the MS care model and has to fit existing standards (e.g., standard value sets for electronic case records). Documents inside the MS case record can be human-readable (e.g., PDF) as well as machine-readable (e.g., Clinical Document Architecture—CDA, FHIR). This dualism enables a staged creation of electronic case records. Projects can start with a defined set of metadata and human-readable documents. Later on, they can introduce higher formalized document standards which also contain machine-readable data.

## Methods

### Design

**Figure 2:**
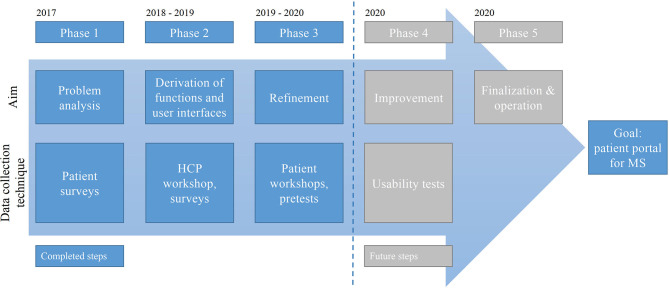
Development phases of IBMS.

Following existing user-centered design approaches and patient-centered participatory design processes, surveys and workshops were conducted, and prototypes were created (21, 30–33). As a guiding research model, the authors applied the five iterative phases of Buurmann (32) which were included in a design science research process (51). The phases guided the iterations of the build-evaluate cycle of the design science research process. The authors initially applied surveys in order to get an understanding of the users (phase 1—problem analysis). The electronic portal solution to be developed addresses the needs of the professional and non-professional side equally in the technical context. According to this, the surveys are used to collect technical requirements. In addition, anticipated needs and technical requirements are to be verified in advance. Both aspects serve as input for the technical analysis as well as the subsequent realization. After phase 1, the authors conducted design workshops with HCPs and patients for designing the graphical user interface (phase 2—derivation of functions and user interfaces, phase 3—refinement) and implemented a prototype of the portal based on an agile software development strategy using Scrum (52). A further step, not described in this paper, is the validation of the portal to consider whether it is deemed as usable, acceptable, and functional and as to whether or not it would eventually need ameliorations. After that, the portal can be finalized (phase 4—improvement, phase 5—finalization and operation) (see Figure 2). These phases are also operationalized by employing Scrum. Due to the iterative approach, the associated obstacles between the treating HCPs and the patients were also identified with the aim of further improving communication between HCPs and patients in the future.

### Procedures: Surveys and Workshops

For the medical concept of the patient portal described here, detailed insights into the treatment process of MS patients were needed. The results of workshops and surveys with HCPs and patients, as well as current findings about the functions of patient portals, were taken into account. It is important to include the requirements of both user groups (MS patients and HCPs), as they have different patient portal demands. Furthermore, they represent the two ends of an information channel. For example, HCPs would certainly like to receive all available clinical information efficiently. Patients may attach more importance to a clear presentation of their examination and treatment appointments. Only by carefully collecting these requirements is it possible to develop a portal that meets the needs of its users and also offers benefits for the providers.

The surveys were not designed as representative surveys from which statements with statistical relevance can be derived. Rather, the surveys had an exploratory character to explore the requirements of patients and HCPs for the patient portal. Consequently, the survey data was analyzed in a purely descriptive manner, no statistical tests of an inductive nature were performed. The results of the workshops were processed and summarized by the project staff.

Prior to the survey and the workshop, each patient was free to withdraw from the survey at any time for any reason without consequences toward the care provided. Because this study involved minimal risk and no personally identifiable information, ethics committee approval was not required.

#### Patients

**Survey (phase 1)**. A patient survey was partly conducted at an information event for MS patients in 2017. Included were MS patients, relatives, and friends of MS patients as well as people interested in MS. Visitors could inform themselves about the project at an information desk provided. In addition to the presentation of the project's objective and informative discussions, the possibility of voluntary and anonymous participation in a survey was pointed out. If necessary, the background of the survey was explained in more detail. For visitors interested in participating, a total of 200 copies of the questionnaire in paper form were available at the information desk. The participating individuals had the opportunity to process the questionnaire on site or submit it later at the next specialist appointment, by mail or electronically. The response in this manner was 41 questionnaires. The remaining copies were laid out by the University Hospital Dresden in the waiting areas for patients and actively handed out by the study assistants of the Multiple Sclerosis Center. Furthermore, questionnaires were distributed in support groups of the German Multiple Sclerosis Society. A total of 210 questionnaires for the evaluation were thus obtained. The questionnaires were sent directly to the electronic data collection system in an anonymized manner.

The questionnaire consists of five separate parts with a total of 17 questions (thereof four open questions) (see Supplementary Material):

1. **Person** (age, MS diseased).
2. **Personal MS** (type of treatment institution, access route, period of MS disease, MS symptoms).
3. **Dealing with information and communication technologies** (use of digital hardware, use for what, use for health, present type of gathering information to MS).
4. **Everyday problems with MS**.
5. **Patient portal** (what use/s should the portal have, requested functions and information).

**Workshop (phase 3)**. Two workshops were conducted in 2019. Included were MS patients and relatives of MS patients interested in using a patient portal; previous knowledge was not necessary. Participants included seven patients and four relatives in the first workshop, and nine patients and one relative in the second. The aim of the workshops was to develop a graphical user interface design based on the input of future users (MS patients and relatives). Requirements were to be developed with the help of different methods (e.g., creative techniques). This was conducted primarily by evaluating previous experiences and user priorities (of the survey). Patients should actively put themselves into possible use cases and evaluate existing concepts accordingly. The workshops were led by two moderators who had experience in the development of medical software as well as expertise in MS.

#### HCPs

**Survey (phase 2)**. HCPs were asked about their ideas of a patient portal by means of an online survey, which was available from October 2018 to July 2019. Included were MS experts as well as HCPs and nurses who treat MS patients. Four hundred invitations were distributed by mail or e-mail and also given to HCPs at congresses and meetings. Participants read and consented to a privacy statement. As an incentive for completing the questionnaire, it was possible to participate in a lottery, for which the participants' data was stored with their consent. The online survey was answered by 22 HCPs and two MS nurses. Only respondents who completed the questionnaire in full were included in the evaluation: after the cleansing of the data set, 16 cases remained including only HCPs and no nurses.

The online survey consisted of 37 questions (with 13 open questions) in five subject areas (see Supplementary Material):

1. **Personal information** [e.g., age, years and context of practicing, number of (MS) patients].
2. **System landscape** (e.g., software products for clinicians and licensed HCPs, usage in medical office and networks (MS), content orientation of software, usage of software and hardware).
3. **Treatment of MS patients** (e.g., MS therapeutic methods, ways and frequency of communication with MS patients, contacts to MS patients and other experts, problems in the treatment of MS patients).
4. **Patient portal** (e.g., requested information and functions for MS patients and HCPs, obstacles and risks of usage).
5. **MS case record** [e.g., requested documents and information for a(n) (inter-institutional) MS case record].

**Workshop (phase 2)**. In a workshop held in 2018, the participants (two HCPs and three developers) developed and prioritized portal functions and discussed their graphic representation. The aim was to work out ideas and requirements for a patient portal from the perspective of medical specialists. Functionalities for the patient as well as initial forms of presentation were the focus of discussion. There were no restrictions with regard to the detailing of individual aspects, so that the workshop could be freely designed in the breadth and depth of the discussion.

## Results

### Surveys and Workshops

#### Patients

**Figure 3:**
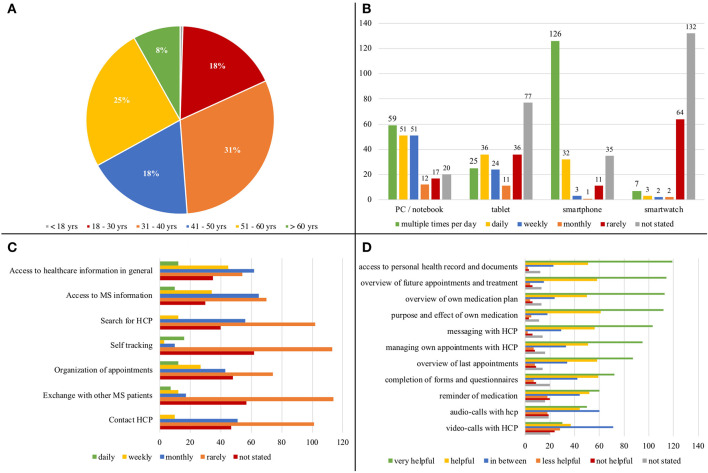
Patient survey participant's… **(A)** age, **(B)** usage of hardware types, **(C)** purpose of technological use, **(D)** assessment of potential portal functions.

**Survey** The majority of the 210 participants were themselves affected by MS (*n* = 182). Additionally, 24 MS patients' relatives and friends as well as four individuals interested in MS also took part in the survey. As close confidants and informal care givers, they enriched the survey results with their positions and experiences. The devices commonly used by the 210 interviewed participants are the smartphone and the PC or notebook (see Figure 3). The majority of respondents are already using these devices to gather information about their health. One of the main problems of the interviewees in everyday life or in dealing with MS is that available information is not understood. Thus, 90% of the patients stated that they could basically imagine the use of such an electronic portal. Among other things, the insight into the patient report and into important documents, an overview of the drugs to be taken including their purpose and effect, as well as an overview of future visits to the HCP were regarded as helpful functions. In addition, the participants surveyed could imagine the following possible functions: communicating with HCPs via the electronic portal solution, networking with other patients, ordering medication, news and event information, and documenting their course of disease. Besides this, there is a need for information regarding MS disease, its treatment, and disease-specific research, the ability to self-help, coping with everyday life and with the illness, as well as concerns regarding legal and official matters.

**Workshop** A patient survey, conducted during the workshops, showed that only two of the 16 patients had already used a patient portal. Despite the limited previous experience, the patients and their relatives showed a high level of interest and openness to use a patient portal. The demands of the patients regarding the functionality of the patient portal (see Figure 3) could be verified in the workshops. As communication-oriented use cases, the patients in the workshops demanded both direct HCP-patient communication and the exchange of information between the care-providers involved in the MS care (e.g., neurologists, general practitioners, and additional therapists). The patients added that documentation sharing between the care provider and the individual should be possible. The graphical visualization of their disease history (symptoms, therapies, treatments) and a diary function (daily documentation of well-being, symptoms, activities, medication) were of great interest. Furthermore, the participants expect to handle administrative procedures such as the application for aids and financial support and to receive information on this issue via the patient portal. The participants await mobile access to their portal data and documents via various devices (e.g., smartphone and tablet). This coincides with the usage of devices resulting from the survey (see Figure 3). In addition, they expect the exchange of data with external digital solutions and services (e.g., activity trackers and apps). As the barrier-free nature of the portal plays a key role, due to the impairments of visual performance, concentration, sensitivity, and motor skills that frequently occur in MS patients, alternatives to these digital devices should be provided (e.g., a print function, voice control, user-specific scaling of the user interface). The high relevance of the portal's accessibility was also demonstrated in the handwritten sketches and paper-based wireframes that the participants designed under the guidance of the moderators. The participants prioritized a clear user interface with low information density, intuitive navigation, fold-out tabs for text input or menu selection, input and search fields with default masks, and user-specific settings (e.g., for the information displayed or scaling of the user interface).

#### HCPs

**Survey** Even if the survey is not representative (Table 1), some interesting aspects can be taken from it; especially since the questionnaire contained many open questions.

**Table 1:** HCP characteristics.

	***N* = 16**
**Age**	
18–50 years	7
>51 years	9
**Specialist**	
Neurologist	12
Double specialist	4
**Years of practice**	
4–10	1
11–25	9
26–40	4
n. s.	2
**Kind of practice**	
Licensed	9
Clinic	7
**Specialized in MS**	
Yes	12
No	4
**Proportion of MS patients/quarter**	
<50%	5
≥50%	11

**Figure 4:**
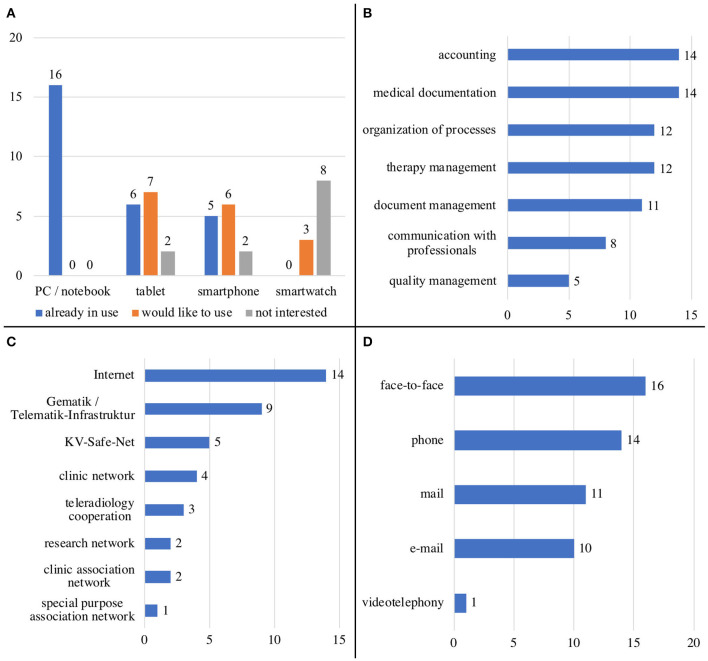
HCP's… **(A)** usage of hardware types, **(B)** usage of software for…, **(C)** connection to…, **(D)** type of HCP-patient-communication.

For the HCPs in the survey, the use of digital hardware seems to already be part of a daily routine or at least conceivable. They use their software products mostly for settlement, medical documentation, and for the organization of processes and therapies. Apps have not been used much so far. HCPs are connected to the internet and also to healthcare-specific networks like the German “Telematik Infrastruktur.” In communication with the patients, the personal conversation has priority, followed by contacts via telephone, mail, and e-mail (see Figure 4).

- **Obstacles in patient treatment**: Some HCPs problematize the data protection regulations and the associated complicatedness of the current type of sending reports via encrypted e-mail. Others believe that communication and coordination between care providers is insufficient. It was also mentioned that there is too little time to talk to the patient, that patients do not pass on information to the HCP themselves, and that the information is sometimes incomplete. Individual HCPs would prefer a consultation via video call as some patients have a long way to travel, and they require more support for patients at home.
- **Patient portal**: As a result of the obstacles when treating MS patients, HCPs request a wide range of functions respectively integrated information for a patient portal, mostly general: information for patients regarding disease (industry independent and neutral, in different versions depending on education, in different languages), typical disease courses, therapy monitoring, medication, tools, remedies, complementary measures, socio-medical, and legal matters as well as contacts (MS practices, outpatient departments and clinics, social authorities, self-help groups). HCPs also request an overview of appointments, the therapeutic process, monitoring, and adverse effects for both HCPs as well as patients. They also ask for possibilities for safe digital communication with patients and other HCPs, as well as care providers (e.g., upload findings, import into hospital management system). Overall, the patient portal should always be up to date, should also be usable on mobile devices, and, if applicable, as an app; access and usability should be easy. For HCPs, the highest risk regarding the patient portal is the privacy policy.
- **MS case record**: For the MS case record, HCPs requested all previous and current relevant findings (laboratory, imaging, medical reports, EDSS etc.). Furthermore, it would be desirable to have data from the socio-medical context within the report, e.g., Barthel index, walking distance, degree of care, provision of aids, sick days (due to MS or other), and gainful activity.

**Workshop** Three topic blocks were discussed and requirements were defined:

- **Access to the portal:** if the patient wishes to use the patient portal, access is granted by the HCP or medical personnel.
- **Dashboard:** The dashboard corresponds to the start page of the portal for the patient after successful login. The following aspects were discussed to be displayed for patients by default: visualized “notification” about (personalized) news about their own health care; and an overview concerning (past and upcoming) appointments, medication, and patient's current tasks (“to dos” e.g., filling out digital questionnaires). For HCPs, the start screen should be designed in such a way that all functions relevant to them can be immediately accessed: a needs-based summary of case and patient information as well as a list of medical “to dos” to be performed during the appointment, graphical presentation of the course of the disease, and the medication process.
- **Functions for patients and HCPs:** In addition to the functions already listed in the dashboard, patients should be able to navigate through a menu to further functions: update profile information as well as store contacts and access rights, view (current and past) medication and request a (follow-up) prescription, view the course of their MS disease in order to track both the temporal occurrence of relapses and changes in course (MS Navigator), view history of MS-related (past and upcoming) appointments, patient-side reporting of illness situation (diary with symptom tracker and pain documentation), and upload or view of medical documents (e.g., MRI, laboratory results, findings, HCP's letters). The medical user should also be able to navigate through a menu to further functions: communication with the patient or consultation with other (MS) experts; view and upload relevant documents; and insight into patient's medication, appointments, and diary.

### Construction of the Patient Portal as a Tool for a Patient-Integrated MS Care Model (Phase 3)

#### Medical Concept

As an organizational framework for digital care provision, a MS care model was developed. The care model consists of organizational structures and processes and references the necessary digital tools. As a process-oriented part, MS specific pathways were developed in the Multiple Sclerosis Center Dresden. These are used as a template for patient-specific pathways that are represented by the patient portal. The MS pathways serve as a conceptual basis for the implementation of the technological patient integration. They are complementary to the organizational structures that are needed to provide inter-organizational MS care. They represent the dynamics of the MS care model (53). The resulting technological solution is a web-based portal which is connected to the existing MSDS^3D^ and a MS case record for access to relevant data. In addition, clinical pathways were operationalized as instruments for MS treatment control and documentation.

**Figure 5:**
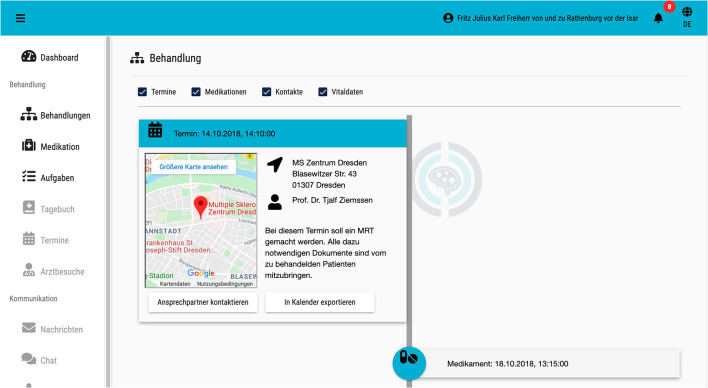
IBMS timeline.

Concretely, the patient portal contains a dashboard for patients with news, a MS Navigator to track temporal occurrence of relapses and changes in course (Figure 5), MS-related (past and upcoming) appointments, (current and past) medication, current tasks and diary (symptom tracker, pain documentation), as well as access to their MS case record. By integrating a diary, patient reported outcomes (PRO) will be taken into account and can be supplemented later by other factors. For HCPs the start page contains a needs-based summary of case and patient information (MS case record) as well as a list of medical “to dos” to be performed during the appointment, and a graphical presentation of the course of the disease and the medication process.

An example scenario should present the functions of IBMS: Mr. X. visits the neurologist in his place of residence to clarify sudden visual disturbances. His neurologist records the suspicion of MS in MSDS^3D^. Mr. X. wishes to be registered in the IBMS, is consequently activated for it by his neurologist via MSDS^3D^, and receives an activation code on site as well as an e-mail with the necessary access information. Via MSDS^3D^ and the central MS case record, the neurologist can also view necessary diagnostic measures and bring in an expert. For diagnostic clarification of the patient's symptoms, MSDS^3D^ is used to arrange and carry out a prompt MRI appointment at the University Hospital. Both the information from the central MS case record and the appointment information as well as the necessary preparatory steps that the patient has to take (e.g., filling out treatment step-related questionnaires) are visible in the timeline of the care portal. Thus, Mr. X. is given the task of filling out a patient admission form in preparation for the examination appointment, which can be done via the IBMS. Following the MRI examination, the data is evaluated by the experts of the University Hospital and the results are reported back to the treating neurologist in real time using the central MS case record. The results can be transferred to the MSDS^3D^. Mr. X. then returns to his treating neurologist. The neurologist receives medical data about the networking between MSDS^3D^ and the central MS case record. The information provided via the care portal (e.g., completed questionnaires) is also transmitted to MSDS^3D^. The experts at the University Hospital can provide the patient with recommendations for action in the form of tasks, information related to the patient-path, and educational materials on the respective path step via the IBMS. Furthermore, the neurologist has the possibility to have a feedback conversation with the expert and to refer to the contents of the central MS case record. Within the IBMS, Mr. X. receives context-sensitive information about his disease. In the process, recommendations for action, made by the experts at the University Hospital, are also taken into account. Mr. X's treatment history can be accessed by a relative if Mr. X grants him the right to do so, which can also be done partially.

### Technological Concept

The patient portal for MS care has been implemented by a modular architecture, which is able to include different external systems. Foundational technologies are Angular (angular.io), Java based on a Wildfly-Server (wildfly.org), and other open source technologies (hapifhir.io, postgresql.org). Consequently, the patient portal can be used in different health information system landscapes. Furthermore, a docker-based (docker.com) implementation eases the portability to new information system landscapes.

**Figure 6:**
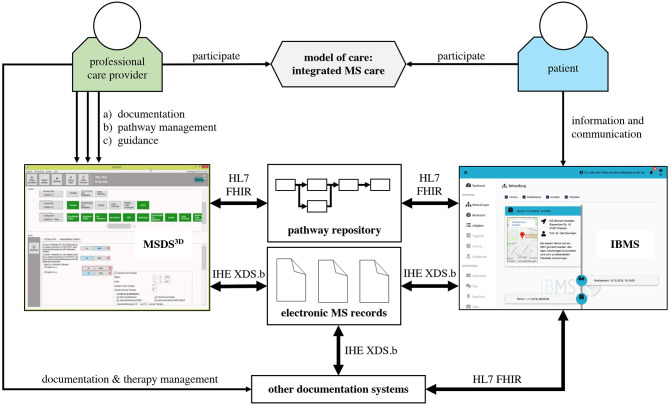
Basic technological concept of IBMS.

In order to achieve the flexibility and interoperability, the technological stack is fully based on the HL7 FHIR interoperability standard (hl7.org/fhir/). A first technological configuration has been built by integrating two main systems: the Multiple Sclerosis Documentation System (MSDS^3D^) and an electronic MS case record (Figure 6). The usage of FHIR enables the patient portal to be a highly integrated but independent system. FHIR enables loose coupling and reduces the efforts for bilateral interface negotiation. Furthermore, due to its technological foundation, the Representational State Transfer (REST)-Paradigm, it is of a high platform independency (26).

A module in the patient portal manages the patient pathways and non-pathway data. Pathway information is implemented by HL7 FHIR resources from the Workflow module. The pathways are stored in a pathway repository. The pathway-relevant data is additionally cached in an integrated FHIR server basic pathway information if external systems are temporarily unavailable. The further technological details for pathway-based application systems can be found in Benedict et al. (26). The patient portal and the MSDS^3D^ furthermore implement FHIR resources for task and questionnaire exchange.

The electronic MS case record is integrated by standard IHE XDS.b-interfaces. The XDS.b standard describes how documents can be shared in an inter-organizational setting. The MS case record implements the standard XDS value sets from the German IHE section (http://www.ihe-d.de/projekte/xds-value-sets-fuer-deutschland/). It is extended by MS-specific document types. In order to achieve interoperability, a hierarchical document type approach is used.

## Discussion

A patient portal for MS patients and HCPs was developed based on the current knowledge of patient portals and clinical pathways, the existing documentation system MSDS^3D^, a MS case record, and the investigation of user needs and concerns. Following Buurmann's five iterative phases, which were integrated into a design science research process, a problem analysis was performed focusing on functions and user interfaces through surveys and workshops with MS patients and HCPs. Based on a user-centered design approach and a patient-centered participatory design process, information and requirements on the professional and non-professional side as well as detailed insights into the treatment process of MS patients were collected with surveys (phase 1). Workshops with HCPs and patients were held for discussing the requirements and design of the graphical user interface (phase 2 and 3). The results of the surveys and workshops highlight that HCPs and patients already use digital hardware and are open to new technologies. Nevertheless, an improved (digital) communication and coordination between care providers is desirable. Both groups require a number of functions for the patient portal. Building on this, the patient portal was refined, and authors implemented a prototype of the portal including MSDS^3D^, an electronic MS case record, and a pathway repository (phase 3). An agile software development strategy was used. A further step, not described here, is the validation of the portal to consider whether it is deemed as usable, acceptable, and functional as well whether it would eventually need ameliorations. Usability tests with patients and HCPs are planned for this (phase 4 and 5).

The innovative digital patient portal has a number of potentially positive impacts for MS patients and their HCPs. It makes decisive contributions to meet the requirements of the enormous diagnostic and therapeutic advances made in neurology. With the help of digital technologies like clinical pathways and case records, the patient portal can help HCPs to better diagnose, monitor long-term, and thus optimally treat individual MS patients. As a result of an optimally adjusted treatment of MS patients, disease progression can be delayed or prevented. Studies using MS-HRS displayed that delaying or preventing disease progression may reduce the societal economic burden of MS (MS-HRS is an easy administrable tool for a holistic assessment of resource utilization from a societal perspective for patients with MS) (54, 55).

The patient portal also offers enormous potential for MS patients, as they face increased challenges from long-term interventions (20). By using the patient portal, MS patients promote their competence and get involved in their treatment process. This can increase the continuity of care and the endurance of MS patients during treatment, as has been seen in other studies (33, 56). Many patients with MS are unable to access health care services for mobility restrictions or lack of locally available health services. The resulting possibilities for coordination between established service providers and expert centers reduce the patient's need to travel to the expert center. Therefore, the patient portal is suitable for use in telerehabilitation. Patients can use the patient portal for individual consultation requests to and from their HCP from home. In this way, HCPs can collect data, monitor patients at home, and consequently change treatment if necessary. As a result, socio-economic costs can be reduced, and patients are thus able to better combine their disease management with their daily social life (20). It is also shown that home-based rehabilitation programs correlate with good patient compliance (20, 57–59). Existing studies show that MS patients displayed improved socialization after telerehabilitation at home compared to the clinical treatment (20). Consequently, the patient portal is a high-quality eHealth solution for all treatment steps from disease-modifying to symptomatic treatment, and also plays an important role when it comes to telerehabilitation (20, 53).

### Perspectives

The implementation of the patient portal highly depends on both technological as well as organizational context factors. First, digital patient portals require a strong integration with medical documentation systems. Proprietary and closed strategies of system providers lead to an insufficient degree of information availability: redundant documentation, interruptions in information flows, and missing transparency of patient status. Therefore, hospitals should move their application systems to support open IT-standards like HL7 FHIR. Secondly, all HCPs need a common understanding of digital patient portals and their management in the inter-sectoral network. This requires a rethinking of their own established processes, behaviors, and cultures. Third, IT-operation of a patient portal is a costly task due to high expectations in security and safety. This needs an adequate refinancing where cost-savings may only appear later in time or indirectly. The reimbursement of costs for the IT operation of the inter-sectoral patient portal must be organized through a multi-stakeholder approach (60).

After successful implementation of the patient portal, the authors see the following perspectives for further development or expansion of the patient portal:

1. **Integration of additional chronic disease patterns:** It is conceivable to also create a patient portal with clinical pathways for Parkinson's disease, diabetes, stroke, or even rare diseases.
2. **Development of a quality manager:** Pathway-based quality indicators can be used to document, monitor, and ideally improve the quality of care for people with MS. They could provide multidimensional quality management tools based on path-based quality indicators for both the patients and the HCPs. For this purpose, the patient portal would be extended by a common path for HCPs and patients. This would make a lasting contribution to patient empowerment, to better integration of care and, above all, to cost reduction through self-management on the part of the patient and quality optimization on the part of the HCP within the framework of recommended MS management (5).
3. **Inclusion of external systems and sensor-based technologies:** As the patient portal allows simple coupling with third-party systems, it is also conceivable to include further external systems, results of remote sensors, wearables, measures of telerehabilitation (e.g., MS Mosaic, Floodlight), and PROs into the patient portal as it is developed (61). Data can be collected continuously at home and not only every three months during a medical consultation (43). Using this approach, more data for the current even more complex management of MS would be available which could be integrated into, as structured for, big data from clinical practices (62).
4. **Development of a privacy manager:** The development of a privacy manager would serve as a tool for patients with chronic illnesses to manage different types of data and data flows within their treatment and to clarify the benefits of these data flows for the patient and to design appropriate security and approval solutions. Through the privacy manager, patients would gain transparency about their own data and its use and can decide for or against the use of their own data for different purposes.
5. **Data Collection using Artificial Intelligence:** Not least, the patient portal is the basis for using artificial intelligence and digital innovations like smart algorithms and expert systems as well as smart communication using the collected well-structured big data from clinical practice. An individualization and constant adaptation of the treatment algorithms by machine learning methods based on data analysis is conceivable. Thus, clinical pathways become adaptable and learn with the patient in the aim of creating personalized pathways. Another idea would be the implementation of chat-bots or avatars, which may help patients access their data in the patient portal.

### Limitations

Because the survey was part of a larger requirements engineering process, the paper does not describe the validation (usability tests with patients and HCPs) of the digital portal. This will be reported in the future. Another important issue to consider, when interpreting the survey, is the low response rate of HCPs. In contrast to the patients, HCPs had little interest in completing the questionnaire. This could mean that doctors may have little interest in a patient portal. They might also associate this with an even greater documentation effort. Perhaps they simply did not have time to fill out the questionnaire, or it was too long or too complex for them. But the low response rate can also mean that an (online) questionnaire is not the right instrument for obtaining HCP's opinions concerning a patient portal. This is all the more likely because the HCPs in the workshop were very interested in setting up a patient portal.

## Data Availability Statement

The raw data supporting the conclusions of this article will be made available by the authors, without undue reservation, to any qualified researcher.

## Ethics Statement

Ethical approval and written informed consent was not required according to local legislation and national guidelines.

## Author Contributions

MB, HS, RK, OM, and TZ conceived the project. MB, IV, RK, OM, and TZ carried out the project. MB, MS, TS, and SF collected data. MB, MS, TS, SF, and IV performed analysis and interpreted results. IV, MB, HS, and TZ wrote the manuscript. All authors reviewed and approved the final manuscript.

## Conflict of Interest

TZ received personal compensation from Biogen, Bayer, Celgene, Novartis, Roche, Sanofi, Teva for the consulting services. TZ received additional financial support for the research activities from Bayer, BAT; Biogen, Novartis, Teva, and Sanofi. RK received personal compensation from Biogen, Bayer, Celgene, Novartis, Roche, Sanofi, Teva for the consulting services. SF and RK were employed by the company MedicalSyn. The remaining authors declare that the research was conducted in the absence of any commercial or financial relationships that could be construed as a potential conflict of interest.
