# Supplementary material for: A Digital Patient Portal for Patients With Multiple Sclerosis
Source: Front Neurol. 2020 May 22;11:400. doi: 10.3389/fneur.2020.00400 (PMC7326091; doi:10.3389/fneur.2020.00400)
Supplement: Supplementary file 1 [file Data_Sheet_1.PDF]

Expertenbefragung - Nutzen von Patientenportalen für die MS-Versorgung

Herzlich willkommen zur Expertenbefragung "Nutzen von Patientenportal für die MS-Versorgung"

Europäische Union

Europa fördert Sachsen.

EFRE

Europäischer Fonds für regionale Entwicklung

Das Universitätsklinikum Carl Gustav Carus Dresden, die Technische Universität Dresden und die Carus Consilium Sachsen GmbH kooperieren im Rahmen des Projektes „Integriertes Betreuungsportal Multiple Sklerose“. Das Projekt wird durch den Freistaat Sachsen und die EU im Zuge des Europäischen Fonds für regionale Entwicklung (EFRE) gefördert.

Ziel des Projekts:

Im Rahmen des Projektes möchten wir ein digitales Patientenportal (Tele-MS-Portal) mit dem Schwerpunkt Multiple Sklerose (MS) entwickeln. Das Portal soll Patienten und Angehörigen die Möglichkeit geben, auf allgemeine Fakten zu MS und insbesondere auf Daten und Informationen zum eigenen Krankheitszustand zuzugreifen. Damit soll die Partizipation der Patienten und Angehörigen im Behandlungsprozess verbessert werden. Durch eine gezielte Vernetzung der beteiligten Mediziner über das Tele-MS-Portal sollen zudem Aufwände reduziert, Kontakthürden abgebaut und die Qualität der Versorgung erhöht werden.

Ziel der Befragung:

Durch Ihre Teilnahme an der Befragung leisten Sie einen entscheidenden Beitrag zur bedarfsgerechten Entwicklung des Patientenportals. Nur durch sorgfältige Erhebung der Anforderungen ist es möglich, ein Portal zu entwickeln, welches den Bedürfnissen seiner Nutzer entspricht und auch auf Seite der Versorger einen Nutzen stiftet.

Daher benötigen wir detaillierte Einblicke in den Behandlungsprozess von MS-Patienten und die damit verbundenen Hürden, welche zwischen behandelndem Arzt und Patient stehen, um die Kommunikation zwischen Arzt und Patient in Zukunft weiter zu verbessern.

Unser Dankeschön für Ihre Teilnahme:

Wenn Sie die Befragung erfolgreich abgeschlossen haben, können Sie sich für eine Verlosung anmelden: Die Carus Consilium Sachsen GmbH stellt hierfür im Rahmen der Projektarbeit **drei Amazon-Gutscheine im Wert zu je 50 EUR** zur Verfügung. Die Anmeldung zur Verlosung ist freiwillig und erfordert die Angabe Ihres Namens und Ihrer E-Mail-Adresse und Telefonnummer. Im Gewinnfall informieren wir Sie telefonisch oder per E-Mail, sie übermitteln uns Ihre Adressdaten, und wir senden Ihnen den Gutschein zu.

Ablauf der Befragung:

Wenn Sie sich zur Teilnahme an der Befragung entscheiden, können Sie direkt dem Link „Weiter“ zu unserer Online-Befragung folgen. Die Bearbeitung des Fragebogens wird ca. 30 Minuten dauern.

Datenschutzerklärung:

Bitte lesen Sie die Datenschutzerklärung sorgfältig durch - für Fragen stehen wir Ihnen gerne zur Verfügung.

1. Zweck und datenerhebende Stelle

Durch Ihre Teilnahme an der **Expertenbefragung - Nutzen von Patientenportalen für die MS-Versorgung** leisten Sie einen entscheidenden Beitrag zur bedarfsgerechten Entwicklung des Patientenportals. Nur durch sorgfältige Erhebung der Anforderungen ist es möglich ein Portal zu entwickeln, welches allen Bedürfnissen seiner Nutzer entspricht und auch auf Seite der Versorger einen Nutzen stiftet.

Daher benötigen wir detaillierte Einblicke in den Behandlungsprozess von MS-Patienten und die damit verbundenen Hürden, welche zwischen behandelndem Arzt und Patient stehen, um zukünftig unkomplizierte und schnelle Kommunikation ermöglichen zu können.

Alleinig für den vorgenannten Forschungszweck sollen anonyme Daten durch den Lehrstuhl für Wirtschaftsinformatik, inbes. Systementwicklung an der TU Dresden (Anschrift: Technische Universität Dresden, Lehrstuhl für Wirtschaftsinformatik, inbes. Systementwicklung, Martin Benedict, 01062 Dresden, E-Mail: martin.benedict@tu-dresden.de) verarbeitet werden (Verarbeitung im Sinne von Erhebung, Speicherung, Veränderung und Nutzung). Hierzu ist eine freiwillige und informierte Einwilligung erforderlich, die bei der Durchführung der Online-Befragung erfolgt.

2. Datenverarbeitung

Die Einwilligungserklärung umfasst die Verarbeitung folgender Daten:

Demographische Daten: Alter, Berufserfahrung, berufliche Position

Betriebliche Daten: Patientenzahl, Therapieverfahren, Daten zu eingesetzten Kommunikationsmitteln, eingesetzte IT-Technik

3. Anonymität

Die Datenerhebung erfolgt anonym, das bedeutet, dass auf Grund der erhobenen Daten keine Rückschlüsse auf Sie gezogen werden können. Wir sichern zudem zu, dass alle Angaben streng vertraulich behandelt werden. Die personenbezogenen Daten (Name, E-Mail, Telefonnummer) werden getrennt von den Umfragedaten gespeichert und könnten nicht mit Ihren Antworten in Verbindung gebracht werden.

4. Datenübermittlung

Die Datenerhebung erfolgt anonym. Es erfolgt keine Übermittlung von personenbezogenen Daten an Dritte.

5. Veröffentlichung

Die Befragungsergebnisse werden in wissenschaftlich üblicher Form veröffentlicht. Wir sichern zu, dass aus den Veröffentlichungen keinerlei Rückschlüsse auf natürliche Personen möglich sind.

6. Freiwilligkeit und Widerruf

Die Teilnahme an der Befragung ist freiwillig. Eine Nichtteilnahme hat keine Folgen. Diese Einwilligung kann jederzeit schriftlich und formlos bei der datenerhebenden Stelle und mit Wirkung auf die Zukunft widerrufen werden. In diesem Fall werden Ihre personenbezogenen Daten gelöscht.

7. Speicherdauer

Alle personenbezogenen Daten werden nach Abschluss des Forschungsprojektes und der Verlosung datenschutzgerecht gelöscht.

8. Datenschutzbeauftragter und Aufsichtsbehörde für den Datenschutz

Sie können sich jederzeit an den Datenschutzbeauftragten der TU Dresden wenden.

9. Auskunftsrecht

Sie haben jederzeit das Recht, Auskunft über die zu Ihrer Person verarbeiteten Daten sowie die möglichen Empfänger dieser Daten, an

die diese übermittelt wurden, zu verlangen. Eine Antwort steht Ihnen mit der Frist von einem Monat nach Eingang des Auskunftsersuchens zu. Bitte beachten Sie, dass dies nur die Daten für die Teilnahme an der Verlosung betrifft. Die Befragungsdaten werden anonym gespeichert und wir können Sie nicht mehr den Teilnehmern zuordnen.

In dieser Umfrage sind 51 Fragen enthalten.

Frage 1.1: Bitte teilen Sie uns Ihre Postleitzahl mit. *

❶ Bitte überprüfen Sie das Format Ihrer Antwort.
Bitte geben Sie Ihre Antwort hier ein:

[ ]

Bitte mindestens drei Stellen angeben.

Frage 1.2 - Sie sind ... *

❶ Bitte wählen Sie eine der folgenden Antworten:
Bitte wählen Sie nur eine der folgenden Antworten aus:

- ○ Allgemeinmediziner
- ○ Neurologe
- ○ Psychiater
- ○ Doppelfacharzt (Neurologe, Psychiater)
- ○ Radiologe
- ○ Urologe
- ○ Anderes [ ]

Frage 1.3 - Wie alt sind Sie? *

❶ Bitte wählen Sie eine der folgenden Antworten:
Bitte wählen Sie nur eine der folgenden Antworten aus:

- ○ 18 - 30 Jahre
- ○ 31 - 40 Jahre
- ○ 41 - 50 Jahre
- ○ 51 - 60 Jahre
- ○ 61 Jahre und älter

Frage 1.4 - Seit wie vielen Jahren praktizieren Sie als Arzt?

❗ Bitte wählen Sie eine der folgenden Antworten:
Bitte wählen Sie nur eine der folgenden Antworten aus:

- ◯ <1 - 3 Jahre
- ◯ 4 - 10 Jahre
- ◯ 11 - 25 Jahre
- ◯ 26 - 40 Jahre
- ◯ Mehr als 40 Jahre
- ◯ Ich praktiziere nicht als Arzt

Frage 1.5 - In welchem Kontext praktizieren Sie momentan? *

❗ Bitte wählen Sie die zutreffenden Antworten aus:
Bitte wählen Sie alle zutreffenden Antworten aus:

- ☐ Niedergelassen - Selbstständig
- ☐ Niedergelassen - MVZ
- ☐ Klinik - ambulant
- ☐ Klinik - stationär
- ☐ Anderes: ____________

Frage 1.6 - Sind Sie auf MS spezialisiert? *

❗ Bitte wählen Sie eine der folgenden Antworten:
Bitte wählen Sie nur eine der folgenden Antworten aus:

- ◯ Ja
- ◯ Nein

Frage 1.7 - Wie viele Patienten (insgesamt, nicht nur MS) behandeln Sie durchschnittlich pro Quartal?

❗ Nur Zahlen dürfen in diese Felder eingegeben werden.

❗ Jede Antwort muss mindestens 0 sein

Bitte geben Sie Ihre Antwort(en) hier ein:

Anzahl Patienten:

Frage 1.8 - Wie viele MS-Patienten behandeln Sie pro Quartal?

❗ Nur Zahlen dürfen in diese Felder eingegeben werden.

❗ Jede Antwort muss mindestens 0 sein

Bitte geben Sie Ihre Antwort(en) hier ein:

Anzahl MS-Patienten

Frage 2.1.1 - Welche der folgenden Softwareprodukte für den klinischen Bereich nutzen Sie momentan?

(Name des Anwendungssystems - Hersteller des Systems, Mehrfachantwort zulässig)

❗ Bitte wählen Sie die zutreffenden Antworten aus:
Bitte wählen Sie alle zutreffenden Antworten aus:

- ☐ CGM Reha - CGM Clinical
- ☐ ClinicCentre - iSolutions Health
- ☐ Clinixx - AMC Holding
- ☐ HoWoS - Gimtec
- ☐ I-Med-One - Telekom
- ☐ ISH-Med - Cerner
- ☐ Medico - Cerner
- ☐ Millennium - Cerner
- ☐ MSDS3D - MedicalSyn
- ☐ Produkte der Nexus AG
- ☐ MMC - Meierhofer
- ☐ ORBIS - Agfa HealthCare GmbH
- ☐ Soarian Clinicals - Cerner
- ☐ Ich verwende kein Produkt für den klinischen Bereich.
- ☐ Sonstiges: ____________

Sofern Sie niedergelassener Arzt sind und trotzdem Softwareprodukte aus diesem Bereich einsetzen, beantworten Sie diese Frage ebenfalls.

Frage 2.1.2 - Welche der Softwareprodukte für den niedergelassenen Bereich nutzen Sie momentan?

(Name des Anwendungssystems - Hersteller des Systems, Mehrfachantwort zulässig)

❗ Bitte wählen Sie die zutreffenden Antworten aus:
Bitte wählen Sie alle zutreffenden Antworten aus:

- [ ] ALBIS - CompuGroup Medical
- [ ] CGM M1 PRO - CompuGroup Medical
- [ ] Data-AL - Data-AL GmbH
- [ ] DURIA - Duria eG
- [ ] Easymed - promedico
- [ ] EL-Elaphe Longisima - Softland GmbH
- [ ] Epikur - Epikur Software
- [ ] MammaSoft - Kassenärztliche Vereinigung Bayerns
- [ ] MSDS3D - medicalsyn
- [ ] MaSc - KV-IT GmbH
- [ ] Medavis RIS - medavis GmbH
- [ ] Medical Office - INDAMED EDV GmbH
- [ ] MEDISTAR - CompuGroup Medical
- [ ] ORBIS - Agfa HealthCare GmbH
- [ ] PSYPRAX - Psyprax GmbH
- [ ] QUINCY WIN - Frey ADV GmbH
- [ ] RadCentre Billing(OPD) - iSolutions Health
- [ ] S3-Win - S3 Praxiscomputer GmbH
- [ ] SAP-Ambulantory Care Management - SAP
- [ ] SMARTY - New Media Company
- [ ] TURBOMED - CompuGroup Medical
- [ ] x.comfort - mediatixx GmbH
- [ ] x.concept - mediatixx GmbH
- [ ] x.isynet - mediatixx GmbH
- [ ] Ich verwende keine Praxissoftware.
- [ ] Sonstiges: ____________

Falls Sie Arzt im klinischen Bereich sind, aber trotzdem eines der Softwareprodukte einsetzen, beantworten Sie diese Frage bitte.

Frage 2.2 - Wie würden Sie die Nutzung der Softwareprodukte in Ihrem Klinik- bzw. Praxisnetzwerk grundlegend charakterisieren?

❗ Bitte wählen Sie die zutreffenden Antworten aus:
Bitte wählen Sie alle zutreffenden Antworten aus:

☐ **Einzelplatzsystem:** Ich habe einen Arbeitsplatz mit einem Rechner, auf dem das Softwareprodukt installiert ist. Einen zentralen Server habe ich nicht.

☐ **Praxisnetzwerk:** Ich habe mehrere verteilte Arbeitsplätze mit mehreren Rechnern, welche vernetzt sind und auf denen das Softwareprodukt installiert ist. Einen zentralen Server habe ich nicht.

☐ **Praxisnetzwerk mit zentralem Server:** Ich habe einen zentralen Server, auf welchem das Softwareprodukt installiert ist. Der Zugriff auf das Softwareprodukt erfolgt über einen oder mehrere Arbeitsplatz-Rechner.

☐ **Web-Lösung/Cloud:** Ich beziehe das System über einen Internetdienst (z. B. Clouddienst) von einem externen Anbieter. Daher ist keine Software direkt auf einem meiner Rechner installiert.

☐ **Praxisnetzwerk mit externem Server:** Ich habe mehrere Rechner, auf denen das Softwareprodukt installiert ist und welche mit dem Internet verbunden sind. Der Server für das Softwareprodukt wird durch einen externen Anbieter außerhalb der Einrichtung betrieben.

☐ **Bildschirmübertragung:** Ich nutze das Softwareprodukt per Bildschirmübertragung auf einem externen Server, welchen ich nicht selbst betreibe. Ich habe daher auf keinem meiner Rechner das Softwareprodukt installiert.

☐ Sonstiges: ____________________

Frage 2.3 - Ist Ihr Softwareprodukt bzw. sind Ihre Softwareprodukte auf die Behandlung von MS-Patienten ausgerichtet? Wenn ja, wie?

(Beispielsweise spezielle Softwareprodukte für MS, eigene Benutzeroberflächen und Funktionen für die MS-Dokumentation oder eigene Parameter zur Erfassung MS-spezifischer Patienteneigenschaften etc.)

❗ Bitte wählen Sie eine der folgenden Antworten:

Bitte wählen Sie nur eine der folgenden Antworten aus:

◯ Nein

◯ Ja (Art und Weise der Anpassung bitte in Kommentarfeld ergänzen)

Bitte schreiben Sie einen Kommentar zu Ihrer Auswahl

Frage 2.4 - Zu welchen Zwecken werden die vorhandenen Softwareprodukte genutzt?

(Mehrfachantwort zulässig)

❗ Bitte wählen Sie die zutreffenden Antworten aus:
Bitte wählen Sie alle zutreffenden Antworten aus:

- ☐ Abrechnung
- ☐ Medizinische Dokumentation
- ☐ Qualitätsmanagement
- ☐ Dokumentenmanagement
- ☐ Organisation der Praxis/Klinikprozesse
- ☐ Therapiemanagement des Patienten
- ☐ Kommunikation mit anderen Leistungserbringern
- ☐ Sonstiges: ____________

Frage 2.5 - Welche Endgeräte nutzen Sie aktuell in der Praxis/Klinik? Welche würden Sie gerne nutzen?

Wenn Sie andere Endgeräte als die vorgegebenen nutzen, bewerten Sie die Zeile "Anderes Gerät" mit "Nutze ich bereits" und beantworten Sie die neu erschienenen Fragen.

Bitte wählen Sie die zutreffende Antwort für jeden Punkt aus:

	**Nutze ich bereits**	**Würde ich gerne nutzen**	**Bin nicht an einer Nutzung interessiert**
**PC/Notebook**	○	○	○
**Tablet**	○	○	○
**Smartphone**	○	○	○
**Smartwatch**	○	○	○
**Anderes Gerät**	○	○	○

Zusatzfrage 2.5.1 - Bitte tragen Sie weitere Geräte ein, die sie nutzen oder gern nutzen möchten.

Beantworten Sie diese Frage nur, wenn folgende Bedingungen erfüllt sind:
Antwort war 'Würde ich gerne nutzen' *oder* 'Nutze ich bereits' bei Frage '14 [B5endgeraet]' (Frage 2.5 - Welche Endgeräte nutzen Sie aktuell in der Praxis/Klinik? Welche würden Sie gerne nutzen? Wenn Sie andere Endgeräte als die vorgegebenen nutzen, bewerten Sie die Zeile "Anderes Gerät" mit "Nutze ich bereits" und beantworten Sie die neu erschienenen Fragen. (Anderes Gerät))

Zusatzfrage 2.5.2 - Bitte bewerten Sie welche der neu hinzugefügten Geräte Sie bereits nutzen oder gerne nutzen würden.

Beantworten Sie diese Frage nur, wenn folgende Bedingungen erfüllt sind:
Antwort war 'Würde ich gerne nutzen' *oder* 'Nutze ich bereits' bei Frage '14 [B5endgeraet]' (Frage 2.5 - Welche Endgeräte nutzen Sie aktuell in der Praxis/Klinik? Welche würden Sie gerne nutzen? Wenn Sie andere Endgeräte als die vorgegebenen nutzen, bewerten Sie die Zeile "Anderes Gerät" mit "Nutze ich bereits" und beantworten Sie die neu erschienenen Fragen. (Anderes Gerät))

Bitte wählen Sie die zutreffende Antwort für jeden Punkt aus:

	**Nutze ich bereits**	**Würde ich gerne nutzen**
**Gerät 1**	◯	◯
**Gerät 2**	◯	◯
**Gerät 3**	◯	◯

Frage 2.6 - Wer ist für die Wartung Ihrer Rechentechnik bzw. IT-Systeme (Rechner, Netzwerk, Softwareprodukte) zuständig? *

❗ Bitte wählen Sie die zutreffenden Antworten aus:
Bitte wählen Sie alle zutreffenden Antworten aus:

- ☐ Arzt selbst
- ☐ ein anderer ärztlicher Kollege
- ☐ Verwaltungsmitarbeiter
- ☐ Schwester/Sprechstundenhilfe
- ☐ Interner IT-Mitarbeiter
- ☐ Externer IT-Dienstleister (nicht APIS/KIS-Anbieter)
- ☐ APIS/KIS-Anbieter
- ☐ Sonstiges: ______

Frage 2.7 - An welches der folgenden Netze ist Ihre Praxis/Ihre Klinik angebunden?

(Mehrfachantwort zulässig)

*

❗ Bitte wählen Sie die zutreffenden Antworten aus:
Bitte wählen Sie alle zutreffenden Antworten aus:

- ☐ Internet
- ☐ KV-Safe-Net
- ☐ Gematik/Telematik-Infrastruktur
- ☐ Teleradiologieverbund
- ☐ Forschungsnetzwerk (z.B. DFN)
- ☐ Zweckverbandsnetzwerk
- ☐ Netzwerk meines Praxissoftwareanbieters
- ☐ Eigenes Ärzteverbundnetzwerk
- ☐ Einbindung in das Netzwerk eines Klinikums
- ☐ Klinikverbundnetzwerk
- ☐ Anbindung an eine regionale eHealth-Plattform
- ☐ meine Praxis / Klinik ist an kein öffentliches Netzwerk angeschlossen
- ☐ Sonstiges: ____________________

Frage 2.8 - Wie wird mit Informationen aus bildgebenden Verfahren umgegangen?

(Mehrfachauswahl zulässig)

❗ Bitte wählen Sie die zutreffenden Antworten aus:
Bitte wählen Sie alle zutreffenden Antworten aus:

- ☐ Bilder werden auf CDs geliefert und mit einem Viewer geöffnet
- ☐ Die Bilder werden in einem eigens dafür vorgesehenen System gespeichert (PACS)
- ☐ Untersuchungsergebnisse werden nur als Bericht und ohne Bildmaterial aufgenommen
- ☐ Die Bilder werden über das Internet übermittelt
- ☐ Sonstiges: ____________________

Frage 3.1 - Welche Therapieverfahren kommen bei der Behandlung Ihrer MS-Patienten zum Einsatz?

(Mehrfachantwort zulässig)

❗ Bitte wählen Sie die zutreffenden Antworten aus:
Bitte wählen Sie alle zutreffenden Antworten aus:

- ☐ Keine medikamentöse Therapie
- ☐ Alemutzumab
- ☐ Azathioprin
- ☐ Cladribin
- ☐ Dimethylfumarat
- ☐ Fingolimod
- ☐ Glatirameracerat
- ☐ Immunglobuline
- ☐ Interferon Beta
- ☐ Methylprednisolon
- ☐ Mitoxantron
- ☐ Natalizumab
- ☐ Ocrelizumab
- ☐ Sonstige Therapie: ________

Frage 3.2 - Gibt es edukative Programme für MS-Patienten in Ihrer Praxis/Klinik?

(Mehrfachantwort zulässig)

❗ Bitte wählen Sie die zutreffenden Antworten aus:
Bitte wählen Sie alle zutreffenden Antworten aus:

- ☐ Nein
- ☐ Ja, Programme der pharmazeutischen Industrie
- ☐ Ja, nicht-industrielle Programme
- ☐ Ja, andere:: ________

Frage 3.3 - Auf welche Art kommunizieren Sie mit Ihren MS-Patienten? Wie läuft die Kommunikation ab?

*(z.B. regelmäßige Vor-Ort Gespräch in der Sprechstunde, Verlaufskontrolle per Telefon durch Sprechstundenhilfe, Übermittlung von Befunden per Post, Terminvereinbarung per E-Mail) **

Bitte geben Sie Ihre Antwort hier ein:

Frage 3.4 - Wie häufig kommunizieren Sie mit einem MS-Patienten durchschnittlich pro Quartal? *

❗ Bitte wählen Sie eine der folgenden Antworten:
Bitte wählen Sie nur eine der folgenden Antworten aus:

- ◯ 0 - 2
- ◯ 3 - 5
- ◯ 6 - 8
- ◯ Mehr als 8

Frage 3.5 - Bitte geben Sie die durchschnittliche Anzahl der Kontakte mit MS-Patienten pro Quartal an (hier ist die Summe aller Kontakte gemeint).

Wenn Sie andere Medien als die vorgegebenen nutzen, tragen Sie bitte in das Feld "Anderes Medium" eine Zahl größer 0 ein und tragen Sie die Medien in die neu erschienenen Fragen ein.

(Stationäre Aufenthalte rechnen Sie dabei bitte jeweils als einen Kontakt pro Aufenthalt)

*

❗ Nur Zahlen dürfen in diese Felder eingegeben werden.
❗ Jede Antwort muss mindestens 0 sein
Bitte geben Sie Ihre Antwort(en) hier ein:

Persönlicher Kontakt (z.B. Sprechstunde, Visite)	
Postalisch (per Briefpost)	
Telefonisch	
E-Mail	
Videotelefonie (z.B. Skype)	
Messenger-Dienste (z.B. WhatsApp)	
Anderes Medium	

Alle Angaben in Kontakte pro Quartal

Zusatzfrage 3.5.1 - Welche anderen Medien benutzen Sie, um mit Ihren Patienten Kontakt aufzunehmen?

Beantworten Sie diese Frage nur, wenn folgende Bedingungen erfüllt sind:
Antwort war gleich oder größer als '1' bei Frage '24 [C5medienkommpat]' (Frage 3.5 - Bitte geben Sie die durchschnittliche Anzahl der Kontakte mit MS-Patienten pro Quartal an (hier ist die Summe aller Kontakte gemeint). Wenn Sie andere Medien als die vorgegebenen nutzen, tragen Sie bitte in das Feld "Anderes Medium" eine Zahl größer 0 ein und tragen Sie die Medien in die neu erschienenen Fragen ein. (Stationäre Aufenthalte rechnen Sie dabei bitte jeweils als einen Kontakt pro Aufenthalt) (Anderes Medium))

Zusatzfrage 3.5.2 - Bitte geben Sie die durchschnittliche Zahl an Kontakten mit MS-Patienten pro Quartal für die zusätzlichen Kommunikationsmedien an.

Beantworten Sie diese Frage nur, wenn folgende Bedingungen erfüllt sind:
Antwort war '1' bei Frage '24 [C5medienkommpat]' (Frage 3.5 - Bitte geben Sie die durchschnittliche Anzahl der Kontakte mit MS-Patienten pro Quartal an (hier ist die Summe aller Kontakte gemeint). Wenn Sie andere Medien als die vorgegebenen nutzen, tragen Sie bitte in das Feld "Anderes Medium" eine Zahl größer 0 ein und tragen Sie die Medien in die neu erschienenen Fragen ein. (Stationäre Aufenthalte rechnen Sie dabei bitte jeweils als einen Kontakt pro Aufenthalt) (Anderes Medium))

❗ Nur Zahlen dürfen in diese Felder eingegeben werden.
Bitte geben Sie Ihre Antwort(en) hier ein:

Kommunikationsmedium 1

Kommunikationsmedium 2

Kommunikationsmedium 3

Frage 3.6 - Wie häufig kontaktieren Sie andere Fachärzte bezüglich einzelner MS-Patienten durchschnittlich pro Quartal? *

Bitte wählen Sie eine der folgenden Antworten:

Bitte wählen Sie nur eine der folgenden Antworten aus:

- 0 - 2
- 3 - 5
- 6 - 8
- Mehr als 8

Frage 3.7 - Bitte geben Sie die durchschnittliche Zahl von Kontakten zu Fachärzten pro Quartal bezüglich der aufgeführten Medien an (bezogen auf alle Kontakte, nicht für einen konkreten Patienten).

Wenn Sie andere Medien als die vorgegebenen nutzen, tragen Sie bitte in das Feld "Anderes Medium" eine "1" ein und tragen Sie die Medien in die neu erschienenen Fragen ein.

Nur Zahlen dürfen in diese Felder eingegeben werden.

Jede Antwort muss mindestens 0 sein

Bitte geben Sie Ihre Antwort(en) hier ein:

Postalisch (per Briefpost)	
Telefonisch	
E-Mail	
Videotelefonie (z.B. Skype)	
Messenger-Dienste (z.B. WhatsApp)	
Fachsoftware (z.B. gemeinsamme elektronische Patientenakte)	
Anderes Medium	

Alle Angaben in Kontakte pro Quartal

Zusatzfrage 3.7.1 - Welche anderen Medien benutzen Sie, um mit anderen Fachärzten Kontakt aufzunehmen?

Beantworten Sie diese Frage nur, wenn folgende Bedingungen erfüllt sind:
Antwort war gleich oder größer als '1' bei Frage '28 [C7kontaktarztmedien]' (Frage 3.7 - Bitte geben Sie die durchschnittliche Zahl von Kontakten zu Fachärzten pro Quartal bezüglich der aufgeführten Medien an (bezogen auf alle Kontakte, nicht für einen konkreten Patienten). Wenn Sie andere Medien als die vorgegebenen nutzen, tragen Sie bitte in das Feld "Anderes Medium" eine "1" ein und tragen Sie die Medien in die neu erschienenen Fragen ein. (Anderes Medium))

Zusatzfrage 3.7.2 - Bitte geben Sie die durchschnittliche Zahl an Kontakten mit Fachärzten für die zusätzlichen Kommunikationsmedien an.

Beantworten Sie diese Frage nur, wenn folgende Bedingungen erfüllt sind:
Antwort war '1' bei Frage '28 [C7kontaktarztmedien]' (Frage 3.7 - Bitte geben Sie die durchschnittliche Zahl von Kontakten zu Fachärzten pro Quartal bezüglich der aufgeführten Medien an (bezogen auf alle Kontakte, nicht für einen konkreten Patienten). Wenn Sie andere Medien als die vorgegebenen nutzen, tragen Sie bitte in das Feld "Anderes Medium" eine "1" ein und tragen Sie die Medien in die neu erschienenen Fragen ein. (Anderes Medium))

❗ Nur Zahlen dürfen in diese Felder eingegeben werden.
Bitte geben Sie Ihre Antwort(en) hier ein:

Kommunikationsmedium 1	
Kommunikationsmedium 2	
Kommunikationsmedium 3	

Frage 3.8 - Welche Hürden existieren bei der Kommunikation mit MS-Patienten?

(Bitte in Stichpunkten oder Sätzen antworten)

Bitte geben Sie Ihre Antwort hier ein:

Frage 3.9 - Inwiefern treffen folgende Probleme auf die Behandlung Ihrer MS-Patienten zu?

Bitte wählen Sie die zutreffende Antwort für jeden Punkt aus:

	Trifft zu	Trifft eher zu	Teils teils	Trifft eher nicht zu	Trifft nicht zu	weiß nicht
**Schlechte Erreichbarkeit der Patienten**	○	○	○	○	○	○
**Späte oder keine Information über auftretende Schübe**	○	○	○	○	○	○
**Späte oder keine Information über aufgetretene Nebenwirkungen**	○	○	○	○	○	○
**Patienten müssen für Rückfragen eine/die/meine Praxis aufsuchen**	○	○	○	○	○	○
**Patienten leiten Informationen nicht selbstständig an behandelnden Arzt weiter**	○	○	○	○	○	○
**Patienten leiten Informationen unvollständig an Arzt weiter**	○	○	○	○	○	○
**Schlechte Compliance der Patienten**	○	○	○	○	○	○
**Patienten haben inhaltliche Verständnisprobleme**	○	○	○	○	○	○

	Trifft zu	Trifft eher zu	Teils teils	Trifft eher nicht zu	Trifft nicht zu	weiß nicht
**Kommunikation mit pflegenden und ergänzenden Leistungserbringern nicht ausreichend**	◯	◯	◯	◯	◯	◯
**Gesamtüberblick über alle Behandlungsaktivitäten (z.B. bei anderen Ärzten) fehlt**	◯	◯	◯	◯	◯	◯
**Patienten haben sprachliche Verständigungsprobleme**	◯	◯	◯	◯	◯	◯

Frage 3.10 - Welche weiteren Probleme, die nicht in Frage 3.9 aufgeführt sind, treten bei der Behandlung von MS-Patienten auf?

(Bitte in Stichpunkten oder Sätzen antworten)

Bitte geben Sie Ihre Antwort hier ein:

Frage 4.1 - Welche Informationen sollten in einem Patientenportal für die MS-Patienten bereitgestellt werden?

(Denken Sie dabei auch an die Anleitung der Patienten in verschiedenen Krankheitsphasen, bitte in Stichpunkten oder Sätzen antworten) *

Bitte geben Sie Ihre Antwort hier ein:

Frage 4.2 - Welche Informationen sollten Ärzte aus einem MS-Patientenportal beziehen können?

(Bitte in Stichpunkten oder Sätzen antworten)

Bitte geben Sie Ihre Antwort hier ein:

Frage 4.4 - Welche Funktionen müsste ein Portal für MS-Patienten aufweisen, damit sowohl Patienten als auch Ärzte es als nützlich betrachten?

(Bitte in Stichpunkten oder Sätzen antworten) *

Bitte geben Sie Ihre Antwort hier ein:

Frage 4.5 - Welche Funktionen würden Sie als besonders wichtig erachten, um die Behandlung von MS-Patienten mit geistigen oder körperlichen Einschränkungen zu erleichtern?

(Sie können Ihre Antwort auf ca. 3 - 5 Funktionen beschränken)

Bitte geben Sie Ihre Antwort hier ein:

Frage 4.6 - Was würde verhindern, dass MS-Patienten ein Patientenportal nutzen?

(Bitte in Stichpunkten oder Sätzen antworten)

Bitte geben Sie Ihre Antwort hier ein:

Frage 4.7 - Welche Risiken sehen Sie in Hinblick auf die Nutzung eines Patientenportals?

(Bitte in Stichpunkten oder Sätzen antworten)

Bitte geben Sie Ihre Antwort hier ein:

Frage 4.8 - Wer sollte Ihrer Meinung nach die Registrierung und die Freischaltung von Daten für den MS-Patienten im Portal vornehmen?

(Mit Registrierung ist Berechtigung des Zugriffs auf alle relevanten und verfügbaren Daten gemeint)

❗ Bitte wählen Sie eine der folgenden Antworten:
Bitte wählen Sie nur eine der folgenden Antworten aus:

◯ Patient registriert sich selbst und hat sofort Zugriff auf seine Daten und Kommunikationswege

◯ Sprechstundenhilfe schaltet Patientinformationen auf elektronische Anfrage durch den Patienten frei

◯ Nur der Arzt sollte Informationen für den Patienten im Zuge einer Sprechstunde freischalten

◯ Andere Methode: ____________

Frage 4.9 - Nehmen Sie an, es gäbe ein Portal für MS-Patienten. Als wie hilfreich würden Sie die folgenden Funktionen für MS-Patienten/für den Arzt einschätzen?

Bitte beachten Sie, dass Funktionen immer solche sind, die durch den Patienten genutzt werden.

Wenn Sie Funktionen hinzufügen möchten, bewerten Sie die "Andere Funktion"-Zeile mit "Sehr hilfreich" für Arzt und Patient und beantworten Sie die neu erschienenen Fragen.

Bitte wählen Sie die zutreffende Antwort für jeden Punkt aus:

	Nutzen für Patient							Nutzen für Arzt					
	sehr hilfreich	ziemlich hilfreich	teils teils	wenig hilfreich	nicht hilfreich	weiß nicht		sehr hilfreich	ziemlich hilfreich	teils teils	wenig hilfreich	nicht hilfreich	weiß nicht
**Patient kann Patientenakte und Dokumente einsehen**	◯	◯	◯	◯	◯	◯		◯	◯	◯	◯	◯	◯
**Patient erhält Überblick über Medikamente, die Patient/-in einnehmen soll**	◯	◯	◯	◯	◯	◯		◯	◯	◯	◯	◯	◯
**Patient kann Rückfragen zum Krankheitsbild an den Arzt senden**	◯	◯	◯	◯	◯	◯		◯	◯	◯	◯	◯	◯
**Patient erhält individuelle Informationen zu Zweck und Wirkung der Medikamente, die er einnehmen soll**	◯	◯	◯	◯	◯	◯		◯	◯	◯	◯	◯	◯
**Patient erhält Erinnerung an Medikamente, die er einnehmen soll**	◯	◯	◯	◯	◯	◯		◯	◯	◯	◯	◯	◯
**Patient erhält Überblick über vergangene Arztbesuche und die dabei erstellte medizinische Dokumentation**	◯	◯	◯	◯	◯	◯		◯	◯	◯	◯	◯	◯

	Nutzen für Patient							Nutzen für Arzt					
	sehr hilfreich	ziemlich hilfreich	teils teils	wenig hilfreich	nicht hilfreich	weiß nicht		sehr hilfreich	ziemlich hilfreich	teils teils	wenig hilfreich	nicht hilfreich	weiß nicht
**Patient erhält Überblick über zukünftige Behandlungen oder Arztbesuche**	○	○	○	○	○	○		○	○	○	○	○	○
**Patient kann beliebige Nachrichten mit behandelnden Ärzten/-innen austauschen**	○	○	○	○	○	○		○	○	○	○	○	○
**Audio-gestützte Gespräche zwischen Arzt und Patient/-in**	○	○	○	○	○	○		○	○	○	○	○	○
**Video-gestützte Gespräche zwischen Arzt und Patient/-in**	○	○	○	○	○	○		○	○	○	○	○	○
**Patient erhält Fragebögen und Formulare und kann diese nach Ausfüllen an Arzt übermitteln**	○	○	○	○	○	○		○	○	○	○	○	○
**Patient kann Arzttermine inhaltlich vorbereiten**	○	○	○	○	○	○		○	○	○	○	○	○
**Patient erhält Aufgaben und Hinweise nach einem Termin**	○	○	○	○	○	○		○	○	○	○	○	○
**Andere Funktion (Antwortmöglichkeit wird eingeblendet)**	○	○	○	○	○	○		○	○	○	○	○	○

Zusatzfrage 4.9.1 - Bitte beschreiben/benennen Sie die von Ihnen erwünschten Funktionen.

Beantworten Sie diese Frage nur, wenn folgende Bedingungen erfüllt sind:
Antwort war 'teils teils' *oder* 'ziemlich
hilfreich' *oder* 'wenig
hilfreich' *oder* 'teils teils' *oder* 'ziemlich
hilfreich' *oder* 'sehr
hilfreich' *oder* 'wenig
hilfreich' *oder* 'sehr
hilfreich' bei Frage '41 [D9funktionenNutzen]' (Frage 4.9 - Nehmen Sie an, es gäbe ein Portal für MS-Patienten. Als wie hilfreich würden Sie die folgenden Funktionen für MS-Patienten/für den Arzt einschätzen? Bitte beachten Sie, dass Funktionen immer solche sind, die durch den Patienten genutzt werden. Wenn Sie Funktionen hinzufügen möchten, bewerten Sie die "Andere Funktion"-Zeile mit "Sehr hilfreich" für Arzt und Patient und beantworten Sie die neu erschienenen Fragen. (Andere Funktion
(Antwortmöglichkeit wird eingeblendet) Beschriftung Nutzen für Patient)) *und* Antwort war 'teils
teils' *oder* 'ziemlich
hilfreich' *oder* 'wenig
hilfreich' *oder* 'teils teils' *oder* 'ziemlich
hilfreich' *oder* 'sehr
hilfreich' *oder* 'wenig
hilfreich' *oder* 'sehr
hilfreich' bei Frage '41 [D9funktionenNutzen]' (Frage 4.9 - Nehmen Sie an, es gäbe ein Portal für MS-Patienten. Als wie hilfreich würden Sie die folgenden Funktionen für MS-Patienten/für den Arzt einschätzen? Bitte beachten Sie, dass Funktionen immer solche sind, die durch den Patienten genutzt werden. Wenn Sie Funktionen hinzufügen möchten, bewerten Sie die "Andere Funktion"-Zeile mit "Sehr hilfreich" für Arzt und Patient und beantworten Sie die neu erschienenen Fragen. (Andere Funktion
(Antwortmöglichkeit wird eingeblendet) Beschriftung Nutzen für Patient))

Zusatzfrage 4.9.2 - Bitte bewerten Sie die von Ihnen hinzugefügten Funktionen hinsichtlich Ihres Nutzens für Patienten und Ärzte.

Beantworten Sie diese Frage nur, wenn folgende Bedingungen erfüllt sind:
((D9funktionenNutzen_D013_0.NAOK (/umfragen/limesurvey/index.php/admin/questions/sa/view/surveyid/283319/gid/1867/qid/45610) == "D910" or D9funktionenNutzen_D013_0.NAOK (/umfragen/limesurvey/index.php/admin/questions/sa/view/surveyid/283319/gid/1867/qid/45610) == "D911" or D9funktionenNutzen_D013_0.NAOK (/umfragen/limesurvey/index.php/admin/questions/sa/view/surveyid/283319/gid/1867/qid/45610) == "D912" or D9funktionenNutzen_D013_0.NAOK (/umfragen/limesurvey/index.php/admin/questions/sa/view/surveyid/283319/gid/1867/qid/45610) == "D913") or (D9funktionenNutzen_D013_1.NAOK (/umfragen/limesurvey/index.php/admin/questions/sa/view/surveyid/283319/gid/1867/qid/45610) == "D920" or D9funktionenNutzen_D013_1.NAOK (/umfragen/limesurvey/index.php/admin/questions/sa/view/surveyid/283319/gid/1867/qid/45610) == "D921" or D9funktionenNutzen_D013_1.NAOK (/umfragen/limesurvey/index.php/admin/questions/sa/view/surveyid/283319/gid/1867/qid/45610) == "D922" or D9funktionenNutzen_D013_1.NAOK (/umfragen/limesurvey/index.php/admin/questions/sa/view/surveyid/283319/gid/1867/qid/45610) == "D923"))

Bitte wählen Sie die zutreffende Antwort für jeden Punkt aus:

	Nutzen für Patient						Nutzen für Arzt				
	sehr hilfreich	ziemlich hilfreich	teils teils	wenig hilfreich	nicht hilfreich		sehr hilfreich	ziemlich hilfreich	teils teils	wenig hilfreich	nicht hilfreich
**Funktion 1**											
**Funktion 2**											
**Funktion 3**											
**Funktion 4**											

Frage 5.1 - Welche Dokumente und Informationen erwarten Sie typischerweise im Kontext der MS-Behandlung von anderen Ärzten und Leistungserbringern?

Bitte geben Sie Ihre Antwort hier ein:

Frage 5.2 - Welche dieser Dokumente und Informationen liegen im Regelfall nicht vor, werden aber von Ihnen benötigt?

(Antwort in Stichpunkten oder Sätzen)

Bitte geben Sie Ihre Antwort hier ein:

Frage 5.4 - Welche weiteren Funktionen sollte es Ihrer Meinung nach bei der einrichtungsübergreifenden MS-Betreuung für die Ärzte geben?

(Antwort in Stichpunkten oder Sätzen)

Bitte geben Sie Ihre Antwort hier ein:

Frage 5.5 - Haben Sie weitere Anmerkungen und Hinweise, die Sie uns mitteilen möchten?

(Antwort in Stichpunkten oder Sätzen)

Bitte geben Sie Ihre Antwort hier ein:

Name:

Bitte geben Sie Ihre Antwort hier ein:

E-Mail-Adresse:

❗ Bitte überprüfen Sie das Format Ihrer Antwort.
Bitte geben Sie Ihre Antwort hier ein:

Telefonnummer für Rückfragen

Bitte geben Sie Ihre Antwort hier ein:

Ich möchte an der Verlosung teilnehmen:

Beantworten Sie diese Frage nur, wenn folgende Bedingungen erfüllt sind:
! is_empty(email (/umfragen/limesurvey/index.php/admin/questions/sa/view/surveyid/283319/gid/1869/qid/45623)) OR ! is_empty(TELEFON (/umfragen/limesurvey/index.php/admin/questions/sa/view/surveyid/283319/gid/1869/qid/45626))

Bitte wählen Sie nur eine der folgenden Antworten aus:

- ◯ Ja
- ◯ Nein

Vielen Dank für Ihre Zeit und die Teilnahme an unserer Befragung!

Übermittlung Ihres ausgefüllten Fragebogens:
Vielen Dank für die Beantwortung des Fragebogens.
