# Supplementary material for: A Digital Patient Portal for Patients With Multiple Sclerosis
Source: Front Neurol. 2020 May 22;11:400. doi: 10.3389/fneur.2020.00400 (PMC7326091; doi:10.3389/fneur.2020.00400)
Supplement: Supplementary file 2 [file Data_Sheet_2.PDF]

Entwicklung eines Patientenportals – Befragung von Patienten und Angehörigen

Wer sind wir?

Wir sind das Universitätsklinikum Carl Gustav Carus Dresden, die Technische Universität Dresden und die Carus Consilium Sachsen GmbH. Zusammen kooperieren wir im Rahmen des Projektes „Integriertes Betreuungsportal Multiple Sklerose“. Das Projekt wird durch den Freistaat Sachsen und die EU im Zuge des Europäischen Fonds für regionale Entwicklung (EFRE) gefördert.

Was wollen wir erreichen?

Im Rahmen des Projektes möchten wir ein digitales Patientenportal (Tele-MS-Portal) mit dem Schwerpunkt Multiple Sklerose (MS) entwickeln. Das Portal soll es Patienten und Angehörigen ermöglichen, Informationen zum eigenen Erkrankungszustand zu beziehen und damit aktuelle Informationen über ihren Krankheitszustand zu erlangen. Damit soll Ihr Einfluss auf die Behandlungsverläufe verbessert und ihre Position als aktiver Partner in der Behandlung gestärkt werden. Durch eine gezielte Vernetzung der beteiligten Mediziner über das Tele-MS-Portal sollen zudem Aufwände reduziert, Kontakthürden abgebaut und die Qualität der Versorgung erhöht werden.

Warum sollten Sie an der Befragung teilnehmen?

Durch die Befragung möchten wir einerseits einen Überblick über Ihre alltäglichen Probleme im Zusammenhang mit der MS-Erkrankung erlangen. Andererseits möchten wir Ihre Vorstellungen und Wünsche zu einem Tele-MS-Portal kennenlernen, um so ein Portal zu gestalten, dass den Bedürfnissen von MS-Patienten entspricht.

Als MS-Patient oder betreuender Angehöriger eines MS-Patienten können Sie stellvertretend an der Befragung teilnehmen, um uns Ihre Erfahrungen, Wünsche und Meinung mitzuteilen. Die Bearbeitung des Fragebogens wird ungefähr <u>20 Minuten</u> dauern. Ihre freiwillige Teilnahme an der Befragung trägt zur bedarfsgerechten Entwicklung des Tele-MS-Portals bei.

Ansprechpartner:

Medizinische Projektleitung

Multiple Sklerose Zentrum
Prof. Dr. Tjalf Ziemssen
Telefon: 0351 - 458 7450
E-Mail: ms@uniklinikum-dresden.de

Projektleitung Systementwicklung

(Für Rückfragen zur Befragung)

Lehrstuhl für Wirtschaftsinformatik,
inbes. Systementwicklung
Martin Benedict, M. Sc.
Telefon: 0351 – 463 32829
E-Mail: martin.benedict@tu-dresden.de

Europäische Union

Europa fördert Sachsen.
EFRE
Europäischer Fonds für regionale Entwicklung

Datenschutzerklärung

Die Befragung wird nach den geltenden gesetzlichen Datenschutzbestimmungen durchgeführt. Die Beteiligung an der Befragung ist freiwillig. Die Befragung wird anonym durchgeführt und die Ergebnisse der Auswertung werden ausschließlich anonymisiert verwendet. Durch eine Verweigerung der Teilnahme entstehen Ihnen keine Nachteile. Die Befragung kann jedoch nur aussagekräftige Ergebnisse liefern, wenn sich möglichst viele Patienten/-innen beteiligen.

Hinweise zur Bearbeitung des Fragebogens

Eine Fragestellung wird durch Ankreuzen der Antwort oder durch Ausfüllen eines Textfeldes beantwortet. Sind Mehrfachantworten möglich, ist dies deutlich gekennzeichnet. Hinweise zur Bearbeitung sind durch *schräge Schrift* hervorgehoben.

Am Ende des Fragebogens bitten wir Sie um die weitere Teilnahme an der Entwicklung des Tele-MS-Portals. Wir würden uns freuen, wenn Sie dem Aufruf folgen und sich in die weitere Entwicklung als aktiver Partner einbringen.

Den Fragebogen können Sie bei uns am Informationsstand zum MS-Tag oder bei Ihrem nächsten Besuch im MSZ abgeben (sofern zeitnah) oder postalisch an folgende Adresse senden:

TU Dresden
Lehrstuhl f. Wirtschaftsinformatik, insbes. Systementwicklung
Patientenbefragung Tele-MS-Portal
Herr Martin Benedict
01062 Dresden

Sie können die Fragebögen auch verschlüsselt und elektronisch über https://securemail.tu-dresden.de (Registrierung erforderlich) an martin.benedict@tu-dresden.de einreichen.

Europäische Union
Europa fördert Sachsen.
EFRE
Europäischer Fonds für regionale Entwicklung

Teil 1 – Ihre Person

Frage 1.1 – Sie sind …

- ☐ … Patient/in mit MS
- ☐ … Angehörige/r einer Patient/in mit MS
- ☐ … Bekannte/r einer Patient/in mit MS
- ☐ Keines davon *(bitte angeben):*

……………………………………………………………………………………………

Frage 1.2 – Wie alt sind Sie?

- ☐ Unter 18 Jahre
- ☐ 18 - 30 Jahre
- ☐ 31 - 40 Jahre
- ☐ 41 - 50 Jahre
- ☐ 51 - 60 Jahre
- ☐ Älter als 60 Jahre

Teil 2 – Ihre Multiple Sklerose-Erkrankung
(als Angehöriger beantworten Sie folgende Fragen bitte stellvertretend)

Frage 2.1 – An welche Einrichtung wenden Sie sich, um ihre MS behandeln zu lassen? *(Mehrfachantwort möglich)*

- ☐ Multiple Sklerose Zentrum des Universitätsklinikums Dresden
- ☐ Anderes MS Zentrum / Neurologisches Zentrum in:

  ……………………………………………

- ☐ Niedergelassener Neurologe
- ☐ Regionales Krankenhaus
- ☐ Hausarzt
- ☐ Sonstige:

  ……………………………………………

Frage 2.1 – Wie weit ist die nächste Einrichtung, an die Sie sich hauptsächlich zur Behandlung von MS wenden, von Ihrem Wohnort entfernt?

- ☐ Weniger als 5 km
- ☐ 5 - 15 km
- ☐ 16 - 35 km
- ☐ Mehr als 35 km

Europäische Union
Europa fördert Sachsen.
EFRE
Europäischer Fonds für regionale Entwicklung

Frage 2.2 – Seit wann sind Sie an MS erkrankt?

- ☐ Weniger als 1 Jahr
- ☐ 1 - 5 Jahre
- ☐ 6 - 10 Jahre
- ☐ 11 - 15 Jahre
- ☐ 16 - 20 Jahre
- ☐ Mehr als 20 Jahre

Frage 2.3 – Durch welche Symptome äußert sich hauptsächlich Ihre MS-Erkrankung? *(Mehrfachantwort möglich)*

- ☐ Müdigkeit
- ☐ Depression
- ☐ Kognitive Störungen
- ☐ Schmerzen
- ☐ Spastik
- ☐ Gehstörungen
- ☐ Sehstörungen
- ☐ Blasenstörungen
- ☐ Darmstörungen

- ☐ Andere Symptome *(bitte angeben):*

…………………………………………………………………………………………

Teil 3 – Ihr Umgang mit Informations- und Kommunikationstechnologien
(folgende Fragen beziehen sich auf Sie als Patient oder Angehöriger direkt)

Frage 3.1 – Wie oft nutzen Sie folgende Endgeräte privat?

	mehrmals täglich	täglich	wöchentlich	monatlich	seltener
PC/Notebook	☐	☐	☐	☐	☐
Tablet	☐	☐	☐	☐	☐
Smartphone	☐	☐	☐	☐	☐
Smartwatch	☐	☐	☐	☐	☐
Anderes Gerät *(bitte angeben)*:	☐	☐	☐	☐	☐

……………………………………………

Frage 3.2 – Nutzen Sie eines oder mehrere der oben genannten Endgeräte, um sich über Ihrer Gesundheit zu informieren?

- ☐ Ja
- ☐ Nein *(bitte weiter mit Frage 3.4)*

Frage 3.3 – Wie oft nutzen Sie eines oder mehrere der oben genannten Endgeräte, zum … ? *(bitte anschließend weiter mit Frage 3.5)*

	mehrmals täglich	täglich	wöchentlich	monatlich	seltener
Abrufen von Informationen zu Gesundheit und Medizin im Allgemeinen	☐	☐	☐	☐	☐
Abrufen von Informationen zu MS	☐	☐	☐	☐	☐
Auffinden von Ärzten/-innen	☐	☐	☐	☐	☐
Aufzeichnen des eigenen Gesundheitszustands („Self-Tracking“)	☐	☐	☐	☐	☐
Organisieren von Behandlungsterminen	☐	☐	☐	☐	☐
Austausch mit anderen MS-Patienten	☐	☐	☐	☐	☐
Kontakt mit Ärzten/-innen aufnehmen	☐	☐	☐	☐	☐
Anderes Thema *(bitte angeben)*: ……………………………………………	☐	☐	☐	☐	☐

Frage 3.4 – Warum nutzen Sie oben genannten Endgeräte nicht oder nicht regelmäßig, um sich über Ihre Gesundheit zu informieren? *(Mehrfachantwort möglich)*

- ☐ Ich bin nicht mit der Technik vertraut.
- ☐ Mir sind keine Angebote zum Thema bekannt.
- ☐ Ich kenne Angebote zum Thema, diese nutzen mir aber nichts.
- ☐ Ich traue den Angeboten zum Thema nicht.
- ☐ Anderer Grund *(bitte angeben)*:

…………………………………………………………………………………………………

Frage 3.5 – Wo informieren Sie sich aktuell über MS? *(Mehrfachantwort möglich)*

☐ Internet ☐ Bücher / Zeitschriften ☐ Arzt
☐ App ☐ Veranstaltungen ☐ Andere Patienten
☐ Andere Quellen oder Details zur getroffenen Auswahl *(bitte angeben)*:

…………………………………………………………………………………………

Teil 4 – Ihre Probleme im Alltag

Frage 4.1 – Inwiefern treffen die folgenden Aussagen zu Problemen im Alltag oder im Umgang mit Ihrer MS-Erkrankung zu?

	trifft zu	trifft eher zu	teils-teils	trifft eher nicht zu	trifft nicht zu
Ich finde keine Informationen über MS.	☐	☐	☐	☐	☐
Ich verstehe die Informationen über MS nicht.	☐	☐	☐	☐	☐
Ich habe keinen Einblick in meine ärztliche Dokumentation, z.B. Befunde.	☐	☐	☐	☐	☐
Ich habe keinen Überblick über meine ärztliche Dokumentation, z.B. Befunde.	☐	☐	☐	☐	☐
Ich verstehe meine ärztliche Dokumentation, z.B. Befunde, nicht.	☐	☐	☐	☐	☐
Ich habe keine Möglichkeiten mich mit Anderen auszutauschen.	☐	☐	☐	☐	☐
Ich habe das Gefühl, dass mehr zur Verbesserung meiner Situation unternommen werden könnte.	☐	☐	☐	☐	☐
Anderes Problem *(bitte angeben)*:	☐	☐	☐	☐	☐

…………………………………………

…………………………………………

Teil 5 – Tele-MS-Portal

Frage 5.1 - Könnten Sie sich grundsätzlich vorstellen, ein Portal (unabhängig von den vorhandenen Funktionen) zu nutzen?

☐ Ja ☐ Nein

Wenn nein, warum nicht *(bitte angeben)*:

……………………………..…………………………

……………………………..…………………………

……………………………..…………………………

Frage 5.2 - Welche Funktionen müsste ein Portal für MS-Patienten aufweisen, damit Sie es als nützlich betrachten? *(bitte in Stichpunkten oder Sätzen antworten)*

Frage 5.3 – Was würde verhindern, dass sie ein Portal mit den in Frage 5.2 genannten Funktionen nutzen? *(bitte in Stichpunkten oder Sätzen antworten)*
Frage 5.4 – Welche Informationen benötigen Sie oder hätten Sie gern, um mit Ihrer MS-Erkrankung gut leben zu können?

Frage 5.5 – Nehmen Sie an es gäbe ein Portal für MS-Patienten. Als wie hilfreich würden Sie die folgenden Funktionen einschätzen?

	sehr hilfreich	ziemlich hilfreich	teils-teils	wenig hilfreich	nicht hilfreich
Patientenakte und wichtige Dokumente einsehen	☐	☐	☐	☐	☐
Überblick über Medikamente, die ich einnehmen soll	☐	☐	☐	☐	☐
Zweck und Wirkung der Medikamente, die ich einnehmen soll	☐	☐	☐	☐	☐
Erinnerung an Medikamente, die ich einnehmen soll	☐	☐	☐	☐	☐
Überblick über vergangene Behandlungen oder Arztbesuche	☐	☐	☐	☐	☐
Überblick über zukünftige Behandlungen oder Arztbesuche	☐	☐	☐	☐	☐
Nachrichten mit behandelnden Ärzten/-innen austauschen	☐	☐	☐	☐	☐
Audio-gestützte Gespräche mit behandelnden Ärzten/-innen führen	☐	☐	☐	☐	☐
Video-gestützte Gespräche mit behandelnden Ärzten/-innen führen	☐	☐	☐	☐	☐
Fragebögen und Formulare von behandelnden Ärzten/-innen ausfüllen	☐	☐	☐	☐	☐
Eigene Termine mit Ärzten/-innen verwalten	☐	☐	☐	☐	☐
Andere Funktion *(bitte angeben)*:	☐	☐	☐	☐	☐

………………………………………………
