# Supplementary material for: A Digital Patient Portal for Patients With Multiple Sclerosis
Source: Front Neurol. 2020 May 22;11:400. doi: 10.3389/fneur.2020.00400 (PMC7326091; doi:10.3389/fneur.2020.00400)
Supplement: Supplementary file 3 [file Data_Sheet_3.PDF]

Expert survey - Benefits of patient portals for MS care

Welcome to the expert survey "Benefits of patient portal for MS care

Europäische Union

Europa fördert Sachsen.
EFRE
Europäischer Fonds für regionale Entwicklung

The University Hospital Carl Gustav Carus Dresden, the Technical University Dresden and Carus Consilium Sachsen GmbH cooperate in the project "Integrated Care Portal Multiple Sclerosis". The Free State of Saxony and the EU as part of the European Regional Development Fund (ERDF) fund the project.

Aim of the project:
Within the scope of the project, we would like to develop a digital patient portal (Tele-MS-Portal) with a focus on Multiple Sclerosis (MS). The portal should give patients and relatives the possibility to access general facts about MS and especially data and information about their own disease state. This should improve the participation of patients and relatives in the treatment process. By means of a targeted networking of the participating physicians via the Tele-MS-Portal, efforts should also be reduced, contact hurdles removed and the quality of care increased.

Aim of the interview:
By participating in the survey, you make a decisive contribution to the demand-oriented development of patient portals. Only by carefully collecting the requirements, it is possible to develop a portal that meets the needs of its users and provides benefits for the providers. Therefore, we need detailed insights into the treatment process of MS patients and the associated obstacles, which between the treating doctor and the patient in order to further improve communication between doctor and patient in the future.

Our thanks for your participation:
If you have successfully completed the survey, you can register for a raffle: The Carus Consilium Saxony GmbH provides three Amazon vouchers worth 50 EUR each for this purpose within the framework of the project work. Registration for the raffle is voluntary and requires you to give your name and your e-mail address and telephone number. If you win, we will inform you by phone or e-mail, you provide us with your address data and we will send you the voucher.

The interview procedure:
If you decide to participate in the survey, you can follow the "Continue" link directly to our online survey. It will take about 30 minutes to complete the questionnaire.

Privacy Policy:
Please read the data protection declaration carefully - we will be happy to answer any questions you may have.

1. Purpose and data collecting body
By participating in the **expert survey - benefits of patient portals for MS care**, you make a decisive contribution to the demand-oriented development of the patient portal. Only by carefully collecting the requirements, it is possible to develop a portal that meets all the needs of its users and provides benefits for the providers.
Therefore, we need detailed insights into the treatment process of MS patients and the barriers that between the treating physician and the patient in order to enable uncomplicated and fast communication in the future.
For the aforementioned research purpose alone, anonymous data shall be stored by the Chair of Business Informatics, esp. Systementwicklung at the TU Dresden (address: Technische Universität Dresden, Chair of Business Informatics, esp. Systementwicklung, Martin Benedict, 01062 Dresden,
E-Mail: martin.benedict@tu-dresden.de) (processing in the sense of collection, storage, modification and use). For this purpose, voluntary and informed consent is required, which can be obtained from the implementation of the online survey.

2. Data processing
The declaration of consent covers the processing of the following data:
- Demographic data: Age, professional experience, professional position
- Operational data: Number of patients, therapy methods, data on means of communication used, IT technology used

3. Anonymity
The data collection is anonymous, which means that no conclusions can be drawn about you based on the data collected. We also assure you that all information will be treated in strict confidence. The personal data (name, e-mail, telephone number) is stored separately from the survey data and could not be linked to your answers.

4. Data transmission
The data collection is anonymous. No personal data is transmitted to third parties.

5. Publication
The survey results are published in the usual scientific form. We assure that no conclusions about natural persons can be drawn from the publications.

6. Voluntary nature and revocation
Participation in the survey is voluntary. Non-participation has no consequences. This consent can be given at any time in writing and be informally revoked at the data collecting agency and with effect for the future. In this case your personal data is deleted.

7. Storage period
All personal data will be deleted in accordance with data protection regulations after the research project and the lottery have been completed.

8. Data protection officer and supervisory authority for data protection
You can contact the data protection officer of the TU Dresden at any time.

9. right of access
You have the right at any time to request information on the data processed concerning you and the possible recipients of these data to whom they have been transmitted. You have the right to receive a reply within one month of receiving the request for information. Please note that this only concerns the data for participation in the raffle. The survey data will be stored anonymously and we cannot assign you to the participants.

There are 51 questions in this survey.
* Minimum information

Question 1.1: Please tell us your postcode. *
Please enter your answer here:

____
Please enter at least three characters.

Question 1.2 - You are... *
Please select only one of the following answers:
- o General Practitioner
- o Neurologist
- o Psychiatrist
- o Double specialist (neurologist, psychiatrist)
- o Radiologist
- o Urologist
- o Other

Question 1.3 - How old are you? *
Please select only one of the following answers:
- o 18 - 30 years
- o 31 - 40 years
- o 41 - 50 years
- o 51 - 60 years
- o 61 years and older

Question 1.4 - How many years have you been practicing as a physician?
Please select only one of the following answers:
- o <1 - 3 years
- o 4 - 10 years
- o 11 - 25 years
- o 26 - 40 years
- o More than 40 years
- o I do not practice as a doctor

Question 1.5 - In what context do you currently practice? *
Please select all applicable answers:
- o Established - Self-employed
- o Established - MVZ
- o Clinic - outpatient
- o Clinic - stationary
- o Other:

Question 1.6 - Are you a specialist in MS? *
Please select only one of the following answers:
- o Yes
- o No

Question 1.7 - How many patients (total, not just MS) do you treat on average per quarter?
Only numbers may be entered in these fields.
Each answer must be at least 0
Please enter your answer(s) here:
Number of patients: _________

Question 1.8 - How many people with MS do you treat per quarter?
Only numbers may be entered in these fields.
Each answer must be at least 0
Please enter your answer(s) here:
Number of patients: _________

Question 2.1.1 - Which of the following software products for clinical area are you currently using?
(Name of the application system - manufacturer of the system; multiple answers allowed)
Please choose the correct answers:
Please select all applicable answers:

- CGM Rehabilitation - CGM Clinical
- ClinicCentre - iSolutions Health
- Clinixx - AMC Holding
- HoWoS - Gimtec
- I-Med-One - Telecom
- ISH-Med - Cerner
- Medico - Cerner
- Millennium - Cerner
- MSDS3D - MedicalSyn
- Products of Nexus AG
- MMC - Meierhofer
- ORBIS - Agfa HealthCare GmbH
- Soarian Clinicals - Cerner
- I do not use any product for clinical use.
- Other:

If you are a resident physician and still use software products from this area you should also answer this question.

Question 2.1.2 - Which of the software products for the established sector are you currently using?
(Name of the application system - manufacturer of the system; multiple answers allowed)
Please select all applicable answers:

- ALBIS - CompuGroup Medical
- CGM M1 PRO - CompuGroup Medical
- Data-AL - Data-AL GmbH
- DURIA - Duria eG
- Easymed - promedico
- EL-Elaphe Longisima - Softland GmbH
- Epicurus - Epicurus Software
- MammaSoft - Association of Statutory Health Insurance Physicians of Bavaria
- MSDS3D - medicalsyn
- MaSc - KV-IT Ltd
- Medavis RIS - medavis GmbH
- Medical Office - INDAMED EDV GmbH
- MEDISTAR - CompuGroup Medical

- ORBIS - Agfa HealthCare GmbH
- PSYPRAX - Psyprax GmbH
- QUINCY WIN - Frey ADV GmbH
- RadCentre Billing(OPD) - iSolutions Health
- S3-Win - S3 practice computer GmbH
- SAP Ambulantory Care Management - SAP
- SMARTY - New Media Company
- TURBOMED - CompuGroup Medical
- x.comfort - mediatixx GmbH
- x.concept - mediatixx GmbH
- x.isynet - mediatixx GmbH
- I do not use any practice software.
- Other:

If you are a doctor in the clinical field, but still use one of the software products, please answer this question.

Question 2.2 - How would you fundamentally characterize the use of the software products in your hospital or practice network?

Please select all applicable answers:

- Single user system: I have a workplace with a computer on which the software product is installed. I do not have a central server.
- Practice network: I have several distributed workstations with several computers which are networked and on which the software product is installed. I do not have a central server.
- Practice network with central server: I have a central server on which the software product is installed. The software product is accessed via one or more workstations.
- Web solution/cloud: I obtain the system via an Internet service (e.g. cloud service) from an external provider. Therefore, no software is installed directly on one of my computers.
- Practice network with external server: I have several computers on which the software product is installed and which are connected to the Internet. An external provider outside the institution operates the server for the software product.
- Screen transmission: I use the software product via screen transmission on an external server, which I do not operate myself. Therefore, I have not installed the software product on any of my computers.
- Other:

Question 2.3 - Is your software product(s) designed to treat people with MS? If so, how? (For example, special software products for MS, own user interfaces and functions for MS documentation or own parameters for recording MS-specific patient characteristics etc.)

Please select only one of the following answers:

- No
- Yes (please add the type and method of adjustment in the comment field)

  Please write a comment to your selection

Question 2.4 - What are the purposes for which existing software products are used?
(multiple answers allowed)
Please select all applicable answers:

- ○ Accounting
- ○ Medical documentation
- ○ Quality Management
- ○ Document management
- ○ Organization of the practice/clinic processes
- ○ Therapy management of the patient
- ○ Communication with other service providers
- ○ Other:

Question 2.5 - Which end devices do you currently use in your practice/clinic? Which one would you like to use?
(If you are using other devices than the default ones, rate the line "Other device" with "Am I already using" and answer the new questions that appear.)
Please select the appropriate answer for each point:

	I already use	I'd like to use	I am not interested in using
PC/notebook	○	○	○
Tablet	○	○	○
Smartphone	○	○	○
Smartwatch	○	○	○
Other device	○	○	○

Supplementary question 2.5.1 - Please list other devices that you use or would like to use
Answer this question only if the following conditions are met:
The answer was 'Would I like to use' or 'Am I already using' in question '14 [B5device]' (Question 2.5 - Which devices do you currently use in your practice/clinic? Which ones would you like to use? If you are using other devices than those specified, please rate the line "Other device" as "I am already using" and answer the new questions. (Other device))

Additional question 2.5.2 - Please rate which of the newly added devices you already use or would like to use.
Answer this question only if the following conditions are met:
Answer was 'Would I like to use' or 'Am I already using' in question '14 [B5 terminal]' (Question 2.5 - Which end devices do you currently use in your practice/clinic? Which would you like to use? If you use other end devices than those specified, evaluate the line "Other device" with "I am already using" and answer the newly published Questions. (Other device))

Please select the appropriate answer for each point:

	I already use	I'd like to use
Device 1	○	○
Device 2	○	○
Device 3	○	○

Question 2.6 - Who is responsible for the maintenance of your computer technology or IT systems (computers, network, and software products)?*
Please select all applicable answers:

- Doctor himself
- another medical colleague
- Administrative employee
- Nurse/consulting hour help
- Internal IT staff
- External IT service provider (not APIS/KIS provider)
- APIS/KIS provider
- Other:

Question 2.7 - To which of the following networks is your practice/clinic connected?*
(multiple answers allowed)
Please select all applicable answers:

- Internet
- KV-Safe-Net
- Gematics/Telematics Infrastructure
- Teleradiology network
- Research network (e.g. DFN)
- Network of special purpose associations
- Network of my practice software provider
- Own network of doctors
- Integration into the network of a hospital
- Hospital network
- Connection to a regional eHealth platform
- my practice / clinic is not connected to a public network
- Other:

Question 2.8 - How is information from imaging modalities handled?
(multiple answers allowed)
Please select all applicable answers:

- Images are supplied on CDs and opened with a viewer
- The images are stored in a dedicated system (PACS)
- Investigation results are only recorded as a report and without pictures
- The images are transmitted via the Internet
- Other:

Question 3.1 - What treatments are used to treat your MS patients?
(multiple answers allowed)
Please select all applicable answers:

- No drug therapy
- Alemutzumab
- Azathioprine
- Cladribine
- Dimethyl fumarate
- Fingolimod
- Glatirameracerate
- Immunoglobulins
- Interferon beta

- Methylprednisolone
- Mitoxantrone
- Natalizumab
- Ocrelizumab
- Other therapy:

Question 3.2 - Are there educational programs for people with MS in your practice/clinic?
(multiple answers allowed)
Please select all applicable answers:

- No
- Yes, pharmaceutical industry programs
- Yes, non-industrial programs
- Yes, others:

Question 3.3 - How do you communicate with your MS patients? How does the communication work?*
(e. g. regular on-site consultation during office hours, progress monitoring by telephone by receptionist, transmission of findings by post, appointments by e-mail)
Please enter your answer here:

Question 3.4 - How often do you communicate with an MS patient on average per quarter? *
Please select only one of the following answers:

- 0-2
- 3-5
- 6-8
- more than 8

Question 3.5 - Please specify the average number of contacts with MS patients per quarter (here the sum is all contacts).*
If you are using media other than the predetermined ones, please enter a number greater than 0 in the "Other Medium" field and enter the media in the newly appearing questions. (Stationary stays please count as one contact per stay)
Only numbers can be entered in these fields. Each response must be at least 0. Please enter your answer(s) here: (All data in contacts per quarter.)

- Personal contact (e.g. consultation, visit) ____________
- Postal (by post) ____________
- Phone ____________
- Video telephony (e.g. Skype) ____________
- Messenger services (e.g. WhatsApp) ____________
- Other medium ____________

Supplementary Question 3.5.1 - What other media do you use to contact your patients?
Answer this question only if the following conditions are met: Answer was equal to or greater than '1' for question '24 [C5mediakommpat]' (Question 3.5 - Please specify the average number of contacts with MS patients per quarter (here is the sum of all contacts). If you are using media other than the specified ones, please enter a number greater than 0 in the "Other Medium" field and enter the media in the newly appearing questions. (Stationary stays please count as one contact per stay) (Other medium))

Supplementary question 3.5.2 - Please enter the average number contacts with MS patients per quarter for the additional communication media.
Answer this question only if the following conditions are met: Answer was '1' for question '24 [C5mediakommpat]' (question 3.5 - Please specify the average number of contacts with MS patients per quarter (here the sum of all contacts is meant). If you are using media other than the ones you have, please enter a number greater than 0 in the "Other Medium" field and enter the media in the newly appearing questions. (Stationary stays, please count as one Contact per stay) (Other medium))

Only numbers can be entered in these fields.
Please enter your answer(s) here:
Communication medium 1 ____
Communication medium 2 ____
Communication medium 3 ____

Question 3.6 - How often do you contact other specialists about individual MS patients on average per quarter?*
Please select only one of the following answers:
- 0-2
- 3-5
- 6-8
- more than 8

Question 3.7 - Please specify the average number of contacts to specialists per quarter with regard to the media listed (relative to all contacts, not for a specific patient).
If you are using media other than the ones you have, please enter a "1" in the "Other Medium" field and enter the media in the newly appearing questions.
Only numbers can be entered in these fields. Each response must be at least 0. Please enter your answer(s) here: (All data in contacts per quarter)
- Postal (by post)
- Phone
- E-mail
- Video telephony (e.g. Skype)
- Messenger services (e.g. WhatsApp)
- Specialist software (e.g. common electronic patient records)
- Other medium

Supplementary question 3.7.1 - What other media do you use to contact other specialists?
Answer this question only if the following conditions are met: Answer was equal to or greater than '1' for question '28 [C7 contact medical media]' (question 3.7 - Please specify the average number of contacts to specialists per quarter with regard to the listed media (based on all contacts, not for a specific patient).
If you are using media other than the ones you have, please enter a "1" in the "Other Medium" field and enter the media in the newly appeared questions. (Other medium))

Supplementary question 3.7.2 - Please enter the average number contacts with specialists for the additional communication media.
Answer this question only if the following conditions are met: Answer was '1' for question '28 [C7 contact medical media]' (question 3.7 - Please enter the average number of contacts to specialists per quarter regarding the listed media (in relation to all contacts, not for a specific patient). If you are using media other than the ones you specify, please enter a "1" in the "Other Medium" field and enter the media in the newly created questions. (Other medium))

Only numbers can be entered in these fields.
Please enter your answer(s) here:
Communication medium 1 ____
Communication medium 2 ____
Communication medium 3 ____

Question 3.8 - What are the hurdles to communication with MS patients?
(Please reply in key points or sentences)
Please enter your answer here:

Question 3.9 - To what extent do the following problems affect the treatment of your MS patients?
Please select the correct answer for each item:

	applies	applies more	part/part	applies less	does not apply	I don't know
poor accessibility of patients	○	○	○	○	○	○
late or no information about occurring relapses	○	○	○	○	○	○
late or no information about side effects	○	○	○	○	○	○
patients must visit a/my practice for queries	○	○	○	○	○	○
patients do not independently pass on information to treating physician	○	○	○	○	○	○
patients conduct information incomplete to doctor	○	○	○	○	○	○
poor patient compliance	○	○	○	○	○	○
patients have content-related comprehension problems	○	○	○	○	○	○
communication with nurturing and complementary service providers is not sufficient	○	○	○	○	○	○
overall overview of all treatment activities (e.g. with other doctors) is missing	○	○	○	○	○	○
patients have language communication problems	○	○	○	○	○	○

Question 3.10 - What other problems that are not listed in question 3.9 occur in the treatment of MS patients?
(Please reply in key points or sentences)
Please enter your answer here:

Question 4.1 - What information should be provided in a patient portal for MS patients?* (Think about the instructions of patients in different phases of the disease)

(Please reply in key points or sentences)

Please enter your answer here:

Question 4.2 - What information should doctors be able to obtain from an MS patient portal?

(Please reply in key points or sentences)

Please enter your answer here:

Question 4.4 - What functions should a portal have for MS patients to make both patients and doctors consider it useful?*

(Please reply in key points or sentences)

Please enter your answer here:

Question 4.5 - What functions would you consider particularly important to facilitate the treatment of MS patients with mental or physical disabilities?

(You can limit your response to about 3 -5 functions)

Please enter your answer here:

Question 4.6 - What would prevent MS patients from using a patient portal?
(Please reply in key points or sentences)
Please enter your answer here:

Question 4.7 - What are your risks in terms of using a patient portal?
(Please reply in key points or sentences)
Please enter your answer here:

Question 4.8 - Who do you think should register and unlock data for the MS patient in the portal?
(Registration means permission to access all relevant and available data)
Please select only one of the following answers:

- Patient registers himself and has immediate access to his data and communication channels
- Consultation assistance unlocks patient information on electronic request by the patient
- Only the doctor should unlock information for the patient during a consultation
- Other method:

Question 4.9 - Suppose there is a portal for MS patients. How helpful would you consider the following functions for MS patients/doctor?
Please note that functions are always those used by the patient.
If you want to add features, rate the "Other Function" line with "Very Helpful" for the doctor and patient and answer the newly appeared questions.
Please select the correct answer for each item:

	**Benefits for Patients**							**Benefits for Doctors**					
	very helpful	quite helpful	part/part	less helpful	not helpful	I don´t know		very helpful	quite helpful	part/part	less helpful	not helpful	I don´t know
patient can view patient records and documents													
patient gets overview of medications that patients should take													
patient can send inquiries about the clinical picture to the doctor													
patient receives individual information on the purpose and effect of the medications he is supposed to take													
patient gets reminder of medication to take													
patient gets an overview of past visits to the doctor and the medical documentation created													
patient gets an overview of future treatments or doctor visits													
patient can exchange any messages with treating physicians													
audio-based conversations between doctor and patient													
video-based conversations between doctor and patient													
patient receives questionnaires and forms and can submit them to doctor after completion													
patient can prepare doctor's appointments in terms of content													
patient receives tasks and hints after an appointment													
other function (response option is displayed)													

Supplementary question 4.9.1 - Please describe/name the functions you want.
(Answer this question only if certain conditions are met.)

Additional Question 4.9.2 - Please rate the features you have added in terms of your benefit to patients and physicians.
(Answer this question only if certain conditions are met.)
Please select the correct answer for each item:

	Benefits for Patients							Benefits for Doctors					
	very helpful	quite helpful	part/part	less helpful	not helpful	I don´t know		very helpful	quite helpful	part/part	less helpful	not helpful	I don´t know
function 1													
function 2													
function 3													
function 4													

Question 5.1 - What documents and information do you expect typically in the context of MS treatment by other physicians and service providers?
Please enter your answer here:

Question 5.2 - Which of these documents and information are usually not available, but are you required?
(Please reply in key points or sentences)
Please enter your answer here:

Question 5.4 - What other functions should there be for physicians in cross-establishment MS care?
(Please reply in key points or sentences)
Please enter your answer here:

Question 5.5 - Do you have any further comments and hints you would like to share with us?
(Please reply in key points or sentences)
Please enter your answer here:

I would like to participate in the raffle:
Please select only one of the following answers:
- Yes
- No

If yes, please enter:
- Name: ____________________
- E-Mail: ____________________
- Telephone number: __________________________

Thank you for your time and participation in our survey!

Europäische Union

Europa fördert Sachsen.
EFRE
Europäischer Fonds für regionale Entwicklung
