# Supplementary material for: A Digital Patient Portal for Patients With Multiple Sclerosis
Source: Front Neurol. 2020 May 22;11:400. doi: 10.3389/fneur.2020.00400 (PMC7326091; doi:10.3389/fneur.2020.00400)
Supplement: Supplementary file 4 [file Data_Sheet_4.PDF]

Development of a patient portal –
Survey of patients and relatives

Who are we?
We are the University Hospital Carl Gustav Carus Dresden, the Technical University of Dresden and Carus Consilium Sachsen GmbH. Together we cooperate within the project "Integrated Support Portal Multiple Sclerosis". The project is supported by the Free State of Saxony and the EU under the European Regional Development Fund (ERDF).

What do we want to achieve?
As part of the project, we would like to develop a digital patient portal (Tele-MS-Portal) with a focus on multiple sclerosis (MS). The portal is intended to enable patients and relatives to obtain information about their own disease condition and thus to obtain up-to-date information about their disease condition. The aim is to improve your influence on treatment processes and to strengthen your position as an active partner in the treatment. Targeted networking of the participating physicians via the Tele-MS portal is also intended to reduce expenses, reduce contact barriers and increase the quality of care.

Why should you take part in the survey?
Through the survey, we would like to gain an overview of your everyday problems in connection with MS disease. On the other hand, we would like to get to know your ideas and wishes for a Tele-MS portal in order to create a portal that meets the needs of MS patients.

As an MS patient or a caregiver of an MS patient, you can take part in the survey on your behalf in order to share your experiences, wishes and opinions with us. The questionnaire will take about 20 minutes to process. Their voluntary participation in the survey contributes to the needs-based development of the Tele-MS portal.

Contact:

Medical Project Management

Multiple Sclerosis Centre

Prof. Dr. Tjalf Ziemssen
Telephone: +49 351-458-7450
E-Mail: ms@uniklinikum-dresden.de

Project Management System Development
(For questions about the survey)
Chair of Business Informatics,
esp. Systementwicklung
Martin Benedict, M. Sc.
Telephone: +49 351-463-32829
E-Mail: martin.benedict@tu-dresden.de

Europäische Union
Europa fördert Sachsen.
EFRE
Europäischer Fonds für regionale Entwicklung

Privacy policy
The survey is carried out in accordance with the applicable statutory data protection regulations. Participation in the survey is voluntary. The survey is carried out anonymously and the results of the evaluation are used exclusively anonymously. You will not be adversely burdened by refusing to participate. However, the survey can only provide meaningful results if as many patients as possible participate.

Notes on the processing of the questionnaire
A question is answered by ticking the answer or by filling in a text box. If multiple responses are possible, this is clearly indicated. Notes for editing are highlighted by *slanted font*.

At the end of the questionnaire, we ask you to continue participating in the development of the Tele-MS portal. We would be pleased if you would follow the call and contribute to the development as an active partner.

You can submit the questionnaire to us at the information stand on the MS day or on your next visit to the MS-Centre (if promptly) or by post to the following address:

TU Dresden
Chair of Business Informatics, esp. Systementwicklung
Patient Survey Tele-MS-Portal
Mr Martin Benedict
01062 Dresden

You can also submit the questionnaires encrypted and electronically via https://securemail.tu-dresden.de (registration required) to martin.benedict@tu-dresden.de

Europäische Union
Europa fördert Sachsen.
EFRE
Europäischer Fonds für regionale Entwicklung

Part 1 - Your Person

Question 1.1 - You are ...

- ... Patient with MS
- ... Relatives of a patient with MS
- ... Known of a patient with MS
- None of them (*please specify*):

....

Question 1.2 - How old are you?

- under 18 years
- 18 - 30 years
- 31 - 40 years
- 41 - 50 years
- 51 - 60 years
- older than 60 years

Part 2 - Your Multiple Sclerosis Disease

(as a relative, please answer the following questions on behalf of the patient)

Question 2.1 - Which institution do you contact to get your MS treated? *(Multiple response possible)*

- Multiple Sclerosis Center of the Dresden University Hospital
- Other MS Center / Neurological center in: ....
- Resident neurologist
- Regional Hospital
- GP
- Other: ....

Question 2.1 – How far is the nearest facility from where you live, which you are mainly contacting for the treatment of MS?

- less than 5 km
- 5 - 15 km
- 16 - 35 km
- more than 35 km

Question 2.2 - Since when have you been diagnosed with MS?

- less than 1 year
- 1 - 5 years
- 6 - 10 years
- 11 - 15 years

- ○ 16 - 20 years
- ○ more than 20 years

Question 2.3 – What are the main symptoms of your MS disease? *(Multiple response possible)*

- ○ Fatigue
- ○ Depression
- ○ Cognitive disorders
- ○ Pain
- ○ Spasticity
- ○ Walking disorders
- ○ Visual impairment
- ○ Bladder disorders
- ○ Intestinal disorders
- ○ other symptoms (please specify): ……………………………………………………………………….

Part 3 - Your Handling of Information and Communication Technologies

(the following questions relate directly to you as a patient or relative)

Question 3.1 - How often do you use the following devices privately?

	several times a day	daily	weekly	monthly	rare
Tablet	○	○	○	○	○
Smartphone	○	○	○	○	○
PC/Notebook	○	○	○	○	○
Smartwatch	○	○	○	○	○
Other device (please specify):	○	○	○	○	○
……………………………………….					

Question 3.2 – Do you use one or more of the above devices to find out about your health?

- ○ Yes
- ○ No (please continue with question 3.4)

Question 3.3 - How often do you use one or more of the above-mentioned devices for...? ***(please continue with question 3.5)***

	several times a day	daily	weekly	monthly	rare
get information about health and medicine in general	○	○	○	○	○
get information about MS	○	○	○	○	○
finding doctors	○	○	○	○	○
record your own state of health ("self-tracking")	○	○	○	○	○
organizing treatment appointments	○	○	○	○	○
exchange with other MS patients	○	○	○	○	○
contact doctors	○	○	○	○	○
other topic (please specify):	○	○	○	○	○
....					

Question 3.4 – Why you do not use the above-mentioned terminals (or not regularly) to find out about your health? ***(Multiple response possible)***

- ○ I am not familiar with the technology.
- ○ I am not aware of any offers on the subject.
- ○ I know offers on the subject, but they do not help me.
- ○ I do not trust the offers on the subject.
- ○ Other reason *(please specify:)* ....

Question 3.5 - Where do you find out about MS? ***(Multiple response possible)***

- ○ Internet
- ○ App
- ○ Books / Magazines
- ○ Events
- ○ Doctor
- ○ Other patients
- ○ Other sources or details of the selection made (please specify):
  ……

Part 4 - Your problems in Everyday Life

Question 4.1 – How do the following statements about everyday problems or how you deal with your MS disease apply?

	applies	applies more	part/ part	applies less	does not apply
I cannot find any information about MS.	○	○	○	○	○
I do not understand the information about MS.	○	○	○	○	○
I have no insight into my medical documentation, e.g. findings.	○	○	○	○	○
I do not have an overview of my medical documentation, e.g. findings.	○	○	○	○	○
I do not understand my medical documentation, e.g. findings.	○	○	○	○	○
I have no options to talk to others.	○	○	○	○	○
I feel that more could be done to improve my situation.	○	○	○	○	○
other problem (please specify):	○	○	○	○	○
……………………………………….					

Part 5 – Tele-MS-Portal

Question 5.1 - Could you basically imagine using a portal (regardless of the existing functions) ?

- ○ Yes
- ○ No
- ○ If no, why not (please specify): ………………………………………………………………………………………

Question 5.2 - What features should a portal have for MS patients to be useful? *(please reply in key points or sentences)*

Question 5.3 - What would prevent you from using a portal with the features mentioned in question 5.2? ***(please reply in key points or sentences)***

Question 5.4 – What information do you need or would you like to have in order to be able to live well with your MS disease?

Question 5.5 - Suppose there is a portal for MS patients. How helpful would you consider the following features?

	very helpful	quite helpful	part/ part	less helpful	not helpful
view patient records and important documents	○	○	○	○	○
overview of the medications I should take	○	○	○	○	○
purpose and effect of the medications I should take	○	○	○	○	○
remembering the medication I should take	○	○	○	○	○
overview of past treatments or visits to the doctor	○	○	○	○	○
overview of future treatments or visits to the doctor	○	○	○	○	○
exchange messages with treating physicians	○	○	○	○	○
audio-based conversations with treating physicians	○	○	○	○	○

	very helpful	quite helpful	part/ part	less helpful	not helpful
video-based conversations with treating physicians	○	○	○	○	○
fill out questionnaires and forms from treating physicians	○	○	○	○	○
manage your own appointments with doctors	○	○	○	○	○
other function (please specify):	○	○	○	○	○
....					

Thank you for your time and participation in our survey!
